# Circular RNA Encoded Amyloid Beta peptides—A Novel Putative Player in Alzheimer’s Disease

**DOI:** 10.3390/cells9102196

**Published:** 2020-09-29

**Authors:** Dingding Mo, Xinping Li, Carsten A. Raabe, Timofey S. Rozhdestvensky, Boris V. Skryabin, Juergen Brosius

**Affiliations:** 1 Max Planck Institute for Biology of Ageing, Joseph-Stelzmann-Strasse 9b, 50931 Cologne, Germany; xinping.li@age.mpg.de; 2 VIB-KU Leuven Center for Brain & Disease Research, KU Leuven, O&N IV Herestraat 49—box 602, 3000 Leuven, Belgium; 3 Medical Faculty, Core Facility Transgenic Animal and Genetic Engineering Models (TRAM), University of Münster, Von-Esmarch-Str. 56, D-48149 Münster, Germany; rozhdest@uni-muenster.de (T.S.R.); skryabi@uni-muenster.de (B.V.S.); 4 Institute of Experimental Pathology, Centre for Molecular Biology of Inflammation (ZMBE), University of Münster, Von-Esmarch-Str. 56, D-48149 Münster, Germany; raabec@uni-muenster.de (C.A.R.); RNA.world@uni-muenster.de (J.B.); 5 Institute of Medical Biochemistry, Centre for Molecular Biology of Inflammation (ZMBE), University of Münster, Von-Esmarch-Strasse 56, D-48149 Münster, Germany; 6 Institutes for Systems Genetics, Frontiers Science Center for Disease-related Molecular Network, West China Hospital, Sichuan University, Chengdu 610212, China

**Keywords:** Alzheimer’s disease (AD), sporadic AD, circular RNA (circRNA), amyloid beta (Aβ), circAβ-a, circRNA translation, Aβ175 polypeptide

## Abstract

Alzheimer’s disease (AD) is an age-related detrimental dementia. Amyloid beta peptides (Aβ) play a crucial role in the pathology of AD. In familial AD, Aβ are generated from the full-length amyloid beta precursor protein (APP) via dysregulated proteolytic processing; however, in the case of sporadic AD, the mechanism of Aβ biogenesis remains elusive. circRNAs are a class of transcripts preferentially expressed in brain. We identified a circRNA harboring the Aβ-coding region of the *APP* gene termed circAβ-a. This circular RNA was detected in the brains of AD patients and non-dementia controls. With the aid of our recently established approach for analysis of circRNA functions, we demonstrated that circAβ-a is efficiently translated into a novel Aβ-containing Aβ175 polypeptide (19.2 KDa) in both cultured cells and human brain. Furthermore, Aβ175 was shown to be processed into Aβ peptides—a hallmark of AD. In summary, our analysis revealed an alternative pathway of Aβ biogenesis. Consequently, circAβ-a and its corresponding translation product could potentially represent novel therapeutic targets for AD treatment. Importantly, our data point to yet another evolutionary route for potentially increasing proteome complexity by generating additional polypeptide variants using back-splicing of primary transcripts that yield circular RNA templates.

## 1. Introduction

Alzheimer’s disease (AD) is one of the most common and devastating forms of dementia. It is associated with the gradual loss of intellectual abilities such as memory and cognition; the progression of AD is accompanied by various behavioral changes [1,2,3,4]. Naturally, as an age-related, neurodegenerative disorder, the greatest known risk factor is increasing age [3,5]. The pathogenesis and clinical manifestation of familial AD is accompanied by the formation of insoluble amyloid beta (Aβ) plaques and neurofibrillary tangles (NFTs) [1,2,3,4,6,7,8,9,10]. Although the actual role of Aβ in AD pathogenesis remains debatable [11], extensive human genetics studies confirmed its strong association with AD disease development [1,12,13]. Aβ peptides are generated from full-length amyloid beta precursor proteins (APP) via sequential proteolytic processing by beta (β) and gamma (γ) secretases [13,14]. Initially, the N-terminal domain of APP is cleaved by β-secretase; subsequently, γ-secretase cleaves the remaining C-terminal fragment of APP (CTF99) to generate Aβ peptides, 36-43 amino acids (aa) in length [14]. Dysregulation of this event is believed to be responsible for Aβ accumulation [14]. Several mutations within the CDS (protein coding sequence) of *APP* were identified and the specific association of these genetic variants with increased accumulation of Aβ were established [12,15,16,17,18,19,20,21,22,23]. Similarly, some diagnostic mutations within presenilin genes, which encode the catalytic subunits of the gamma secretase complex, are linked to increased Aβ levels [24,25,26,27,28,29,30,31,32]. Subsequent polymerization of Aβ peptides leads to oligomers, which in turn aggregate to insoluble amyloid plaques. This process is believed to cause tau hyperphosphorylation. The resulting formation of neurofibrillary tangles initiates a complex cascade of cellular reactions that ultimately lead to neuronal death [1,2,23,26,29,33,34].

However, pathogenesis mediated by genetic mutations is associated exclusively with familial AD, which accounts for only 1–5% of all cases worldwide [1,2]. In fact, the most common form of AD is the “sporadic” variant. This specific form of AD prevails in patients aged 65 or older and unlike familial AD, no specific mutations in *APP* and presenilin genes were identified. However, apart from these striking genetic differences, both forms are characterized by the overproduction of Aβ plaques in the brain [2,11,12,14,35,36]. Although beta-secretase (BACE1) expression and its enzymatic activity were reported to increase in most sporadic AD patients, APP full-length protein and γ-secretase proteolytic activity remained unchanged (compared to non-dementia controls) [37,38]. In addition, extensive analysis of transgenic mouse models for monitoring pathophysiological consequences of human wildtype (unmutated) APP overexpression did not reveal signs of increased Aβ plaque formation in the brain [39,40,41,42]. It is important to note that in most *APP* transgenic mouse models, overexpression of *APP* cDNA was used, thereby excluding circRNA formation [39]. Impaired clearance of Aβ is one proposed mechanism underlying Aβ accumulation [43]; yet, the mechanism of Aβ production in sporadic AD remains largely elusive. However, in vivo data suggest an alternative pathway and/or precursor that leads to Aβ peptide generation.

Circular RNAs (circRNAs) are abundant processing products from primary transcripts, chiefly derived from protein coding genes [44,45,46]. They are the result of heterogeneous nuclear RNA (hnRNA)/pre-mRNA back-splicing and, as such, represent covalently closed circles, which are devoid of RNA cap structures or terminal poly(A) tails [45,47,48]; these features render them substantially different from their corresponding linear mRNA counterparts [45]. Previous analysis revealed that circRNA formation is most prominent in the brain [49,50,51]. Interestingly, in several organisms, circRNAs are regulated in an age-dependent manner [51,52,53,54,55,56]. This might suggest that circRNAs play regulatory roles during aging and are potentially responsible for the development of age-associated neurodegenerative diseases [51,52,53,54,55,56]. More importantly, recent studies demonstrate that certain circRNAs encode proteins, suggesting an essential biological role in addition to that of linear mRNA [57,58,59,60].

We detected an APP primary transcript derived circRNA from human brain samples of AD patients and non-dementia controls. Since the circRNA contains the corresponding Aβ coding sequence, it is referred to as circAβ-a. Recently, we exploited ‘intron-mediated enhancement’ (IME), and successfully established a method for the investigation of circRNA translation in cell culture [57]. We demonstrated that circAβ-a serves as a template for the synthesis of a novel Aβ-containing polypeptide variant. Notably, the resulting product is further processed into Aβ peptides in human embryonic kidney 293 (HEK293) cells, which represents a well-established cellular model in AD research [61,62].

Our findings established an alternative pathway to Aβ biogenesis in human cells via circRNA translation. The resulting polypeptide variant features APP CDS exons 14–17 and an exon 14-derived C-terminus translated out-of-frame that the canonical mRNA could not have generated.

## 2. Materials and Methods

### 2.1. circAβ-a Identification via RT-PCR and Sequencing

The *APP* gene-derived circRNAs were amplified via RT-PCR with specific ‘divergent’, i.e., head-to-head oriented primers targeting protein coding exon 17 of the *APP* gene. One µg of total human RNA samples from the prefrontal cortex of AD patients and healthy donors (obtained from the London Neurodegenerative Diseases Brain Bank, London, UK) were used as template. The studies involving human brain samples were approved by the ethical committees of Leuven University and UZ Leuven. cDNA synthesis was performed with SuperScript™ II Reverse Transcriptase (18064022, Invitrogen, Carlsbad, CA, USA) with random hexamer primers, according to the manufacturer’s recommendations. PCR was performed with Phusion High-Fidelity DNA Polymerase (M0530L, NEB, Ipswich, MA, USA) for 40 cycles according to the manufacturer’s recommendations.

For RNase R treatment, about 15 µg of total human RNA from the prefrontal cortex was treated with 10 units of RNase R (RNR07250, Epicentre, Madison, WI, USA) for 1 h at 37 °C and purified by phenol-chloroform extraction. For subsequent cDNA synthesis and PCR amplifications, 250 ng of the resulting RNA samples were utilized. PCR products were purified by PCR purification kit (28104, QIAGEN, Hilden, Germany) and subsequently verified by Sanger sequencing.

### 2.2. Plasmid Construction

Human hsa_circ_0007556 (circBase), is referred to as circAβ-a in this study [63]. Exons 14, 15, 16, and 17 of the *APP* gene (GRCh37/hg19, chr21:27264033–27284274) representing circAβ-a were inserted into pCircRNA-DMo vectors as described previously [57] to generate pCircRNA-DMo-Aβ-a. As positive control for Aβ175 protein expression, the cDNA containing its open reading frame (ORF) was inserted into the pCMV-MIR vector (OriGene) yielding pCMV-Aβ175-cDNA. A FLAG tag sequence (DYKDDDDKPP) was added to pCircRNA-DMo-Aβ-a to generate pCircRNA-DMo-Aβ-a-FLAG. Recombinant plasmids were purified with EndoFree Plasmid Maxi Kit (12362, QIAGEN, Hilden, Germany). Oligonucleotide sequences and further details are provided in Table S1. All plasmids were verified by restriction endonuclease digestions and Sanger sequencing.

### 2.3. Cell Culture and Plasmid DNA Transfection

Cell culture and plasmid DNA transfection in HEK293 or mouse neuroblastoma N2a cells (N2a cells) were performed as previously described [57]. In addition, 50 µM α-secretase ADAM10 inhibitor GI254023X (SML0789, Sigma-Aldrich, St. Louis, MO, USA), 10 µM β-Secretase Inhibitor IV-CAS 797035-11-1-Calbiochem (565788-1MG, Millipore, Burlington, MA, USA), and 50 µM γ-Secretase Inhibitor (Begacestat, PZ0187, Sigma-Aldrich, St. Louis, MO, USA) were added to the cell culture for 24 h.

### 2.4. Total RNA Isolation and qRT-qPCR

Total RNA from HEK293 cells and human brain prefrontal cortex was isolated with the TRIzol reagent (15596026, Invitrogen, Carlsbad, CA, USA) according to the manufacturer’s recommendations. cDNA synthesis and qRT-PCR were performed as previously described [57]. Details of qRT-PCR oligonucleotides are provided in Table S1.

### 2.5. Northern Blot Hybridization

Northern blot hybridizations were conducted with the NorthernMax™ Kit (AM1940, Ambion, Austin, TX, USA) as previously described [57]. In brief, 15 µg total RNA from HEK293 cells were separated on 5% Criterion™ TBE polyacrylamide gels (3450048, Bio-Rad, Hercules, CA, USA) and transferred to positively charged nylon membranes (AM10100, Ambion, Austin, TX, USA). Hybridization was performed with a 5′ P^32^-labeled DNA oligonucleotide overnight at 42 °C (NB-R1: 5′ CCCACCATGAGTCCAATGATTGCACCTTTGTTTGAACCCACATCTTCTGCAAAGAACACC 3′). Membranes were washed at 42 °C according the manufacturer’s recommendations (see NorthernMax™ Kit (AM1940, Ambion, Austin, TX, USA) for details). For RNase R treatment, 15 µg total RNA were digested with 10 units of RNase R (RNR07250, Epicentre, Madison, WI, USA) for 1 h at 37 °C; RNAs were separated by gel electrophoresis and analyzed by Northern blot hybridization as described above.

### 2.6. Western Blot Analysis

Protein lysates were prepared with RIPA Buffer (50 mM Tris-HCl pH 8.0, 150 mM NaCl, 1% (*v*/*v*) NP40, 0.1% (*w*/*v*) SDS, 0.5% (*w*/*v*) Na-deoxycholate, protease inhibitor [4693132001, Roche, Basel, Switzerland], and PhosSTOP Phosphatase Inhibitor [4906845001, Roche, Basel, Switzerland]). Aliquots representing 40 µg total protein were separated on 4 to 20% Novex Tris-Glycine Mini Gels (XP04205BOX, Invitrogen, Carlsbad, CA, USA) or 4–20% Criterion™ TGX™ Precast Midi Protein Gel (5671094, Bio-Rad, Hercules, CA, USA) and transferred to 0.2 µm nitrocellulose membranes (GE10600002, Amersham, Little Chalfont, UK). Immunoblotting was performed with anti-β-amyloid antibody (clone WO2, MABN10, Sigma-Aldrich, St. Louis, Missouri, USA) (β-Amyloid [D54D2] XP^®^ Rabbit mAb #8243, Cell Signaling Technology, Danvers, MA, USA), monoclonal ANTI-FLAG M2 antibody (F3165, Sigma-Aldrich, St. Louis, MO, USA), and anti-β-actin (A5441, Sigma-Aldrich, St. Louis, Missouri, USA) antibody as previously described [57]. A polyclonal antibody against Aβ175 (Anti-Aβ175) was raised in rabbit with the unique peptide (CFRKSKTIQMTSWPT) as antigenic determinant by GenScript (Piscataway, NJ, USA). Aβ42 peptide (A9810, Sigma-Aldrich, St. Louis, MO, USA) was dissolved in DMSO. Quantitative analysis was performed with ImageJ (NIH).

### 2.7. Immunoprecipitation–Mass Spectrometry (IP-MS) of circAβ-a Derived Protein

pCircRNA-DMo-Aβ-a or pCircRNA-DMo-Aβ-a-FLAG was transfected into HEK293 or N2a cells with secretase inhibitors for 24 h as previously described. Cells were collected and lysed in RIPA buffer [57]. Immunoprecipitations were performed with anti-β-Amyloid antibodies 6E10 and 4G8 (803001, 800701, BioLegend Inc., San Diego, CA, USA) bound to Dynabeads™ Protein A, G (10002D, 10004D, Invitrogen, Carlsbad, CA, USA). Immunoprecipitated proteins were digested on beads with trypsin (V5280, Omega, Biel/Bienne, Switzerland). Anti-Aβ175 was used for immunoprecipitation followed by mass spectrometry of circAβ-a-derived peptides from human brain extract in RIPA buffer. Further details of the procedure and mass spectrometry have been described previously [57].

### 2.8. Immunoprecipitation-Western Blotting (IP-WB) of Aβ Peptides

Aβ peptide detection was performed through immunoprecipitation of conditioned medium (CM), followed by Western blot analysis as previously described [64]. In brief, HEK293 cells transfected with pCircRNA-DMo-Aβ-a or empty vector (pCircRNA-DMo) were cultured in serum-free medium overnight. Then, CM was prepared with protease inhibitor [4693132001, Roche, Basel, Switzerland] and PhosSTOP Phosphatase Inhibitor [4906845001, Roche, Basel, Switzerland]); then it was pre-cleaned with protein A/G beads (Dynabeads™ Protein A, 10002D, Dynabeads™ Protein G, 10004D, Invitrogen, Carlsbad, CA, USA). Immunoprecipitation was conducted with a mixture of Aβ antibodies (6E10, 4G8, BioLegend Inc., San Diego, CA, USA) and protein A/G beads. Precipitated peptides were subsequently dissolved in SDS loading buffer and analyzed by Western blot with an antibody derived against Aβ (D54D2, Cell Signaling Technology, Danvers, MA, USA).

### 2.9. Internal Ribosome Entry Site (IRES) Analysis

The circAβ-a sequence was submitted to IRESbase to detect matches with previously established IRES [65].

## 3. Results

### 3.1. Presence of circAβ-a in the Brain of AD Patients and Non-Dementia Controls

**Figure 1:**
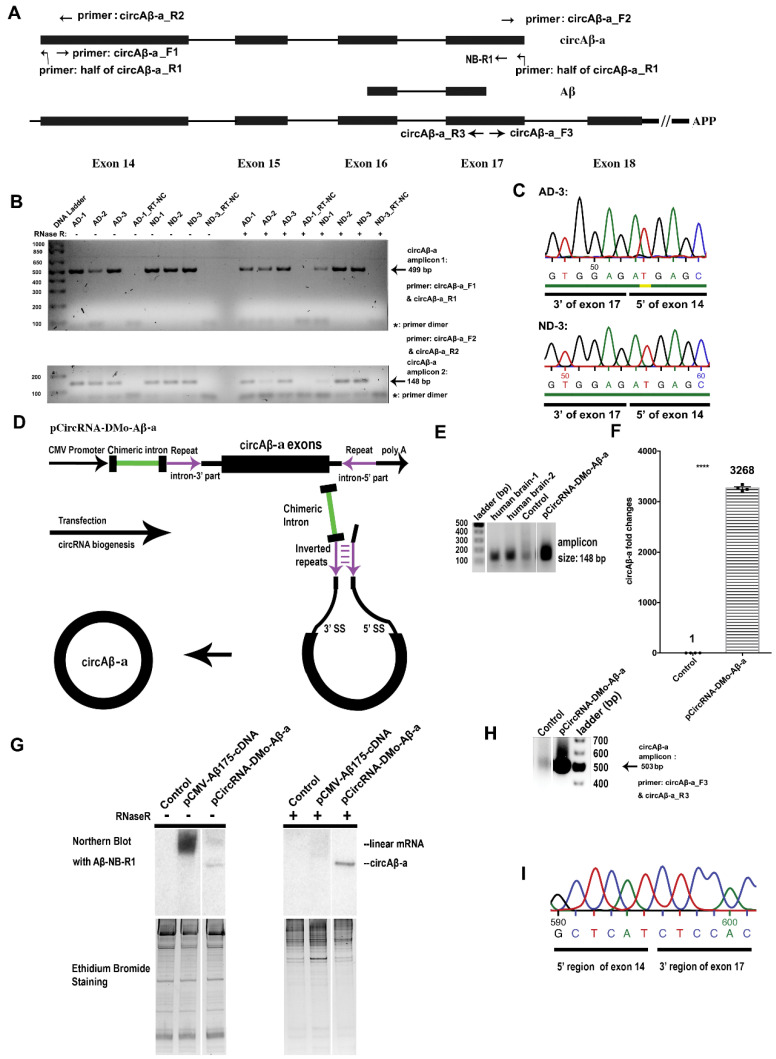
Identification of circAβ-a formation in the human brain and its transient overexpression in HEK293 cells. (**A**). Position of circAβ-a within the amyloid beta precursor protein (APP) coding region. The Aβ sequence is indicated as reference and maps to protein coding exons 16 and 17. circAβ-a consists of exons 14, 15, 16, and 17. Primer positions for RT-PCR and Northern blot hybridization are indicated. (**B**). Agarose gel electrophoresis (1%) of circAβ-a RT-PCR products. Amplifications were performed with a set of divergent oligonucleotide primers (circAβ-a_F1 and circAβ-a_R1, see upper panel) on human prefrontal cortex total RNA from Alzheimer’s disease (AD) and non-dementia (ND) controls as templates. Primer pair circAβ-a_F2 and circAβ-a_R2 was employed for circAβ-a amplification by qRT-PCR; the resulting PCR products were also analyzed by 1% agarose gel electrophoresis (see lower panel). An RNase R digestion step was incorporated for the enrichment of circular RNA. RT-NC: The negative control for reverse transcription, i.e., samples without reverse transcriptase step. (**C**). Sequencing of RT-PCR products (see B for details), confirms the fusion of exon 17 (3′ end) to exon 14 (5′ end), representing the actual junction region of circAβ-a. cDNAs were generated with RNase R pre-treated total RNA of AD-3 and ND-3 samples with hexamer primers. PCR was performed with divergent primer pair (circAβ-a_F2 and circAβ-a_R2). (**D**). circAβ-a overexpression constructs. Figure D is modified from [57]. Our circRNA formation strategy based on intron-mediated enhancement (IME) is schematically depicted (**E**). RT-PCR amplification confirmed circAβ-a formation in human brain samples and recombinant HEK293 cells overexpressing circAβ-a. Control represents mock transfection with pCircRNA-DMo empty vector, pCircRNA-DMo-Aβ-a depicts circAβ-a formation after transfection with pCircRNA-DMo-Aβ-a (i.e., the circAβ-a expression construct). (**F**). Quantification of circAβ-a levels in HEK293 cells. Control represents mock transfection with empty vector (pCircRNA-DMo), Fold changes (*y*-axis) and Student’s T-tests were performed in comparison to the control sample, ****, *p* ≤ 0.0001, *n* = 4. Mean ± SEM of Control: 1.019 ± 0.1216, *n* = 4; Mean ± SEM of pCircRNA-DMo-Aβ-a: 3268 ± 27.22, *n* = 4. (**G**). Northern blot analysis of circAβ-a formation in HEK293 cells. The pCMV-Aβ175-cDNA expression vector harbors the linear ORF corresponding to circAβ-a under the control of the CMV promoter for expression;- depicts untreated samples; + depicts RNase R treated samples, ethidium bromide staining of the 15 µg total RNA in native 5% polyacrylamide gels served as loading control. (**H**). Agarose gel electrophoresis (1%) for circAβ-a RT-PCR products (503 bp). RT-PCR reactions were performed with primers circAβ-a_F3 and circAβ-a_R3. (**I**). Sanger sequencing for the Aβ junction region. PCR products (see above) were cloned and sequenced showing the circAβ-a junction region representing RT-PCR products from the pCircRNA-DMo-Aβ-a transfection. The results also confirmed that circAβ-a was otherwise identical to spliced CDS exons 14, 15, 16, and 17 of the APP gene (data not shown).

Mechanisms of Aβ production in sporadic AD remain elusive [43]. In search of alternative pathways to Aβ biogenesis, we analyzed circRNAs containing Aβ coding regions. circRNA hsa_circ_0007556 harbors APP protein coding exons 14 to 17, is 524 nt in length, and has been detected independently in several RNA high-throughput sequencing datasets [44,49,63,66]. We investigated whether a potential protein product of hsa_circ_0007556 (here referred to as circAβ-a) can constitute an alternative route to Aβ peptide generation. For the detection of circAβ-a in various human brain samples, we performed RT-PCR with two pairs of divergent primers (circAβ-a-F1 and circAβ-a-R1, Figure 1A,B). To account for the interindividual variance of circAβ-a formations, we included six independent samples (prefrontal cortex total RNA) representing AD patients (three) and (ND) non-dementia controls (three), respectively (Figure 1B). In our analysis, reverse primer circAβ-a-R1 mapped to the splice junction region of circAβ-a; this design ensured the specificity of our PCR reaction (Figure 1A). In addition, due to the divergent orientation of our primer pair, this PCR assay required circular molecules as a template. We detected only one amplification product of the expected size (499 bp), i.e., for all AD and non-dementia control samples (Figure 1B). To increase specificity, an RNase R pre-treatment of total RNA samples was incorporated, which enabled the digestion of linear RNA but left circular molecules unaffected. As anticipated, we again detected an amplification product of the same size (499 bp). Sanger sequencing of the resulting PCR amplicon confirmed that the circRNA contained only APP protein coding exons 14–17 and no intronic sequences (Supplementary data-1). These results were confirmed with a second pair of oligonucleotide primers (circAβ-a-F2 and circAβ-a-R2, Figure 1A,B). Migration of the resulting amplicons in agarose gel electrophoresis revealed PCR products of ~150 bp, which is in agreement with the expected amplicon length of 148 bp. Again, the same product was obtained by RNase R treatment prior to RT-PCR assays, once more confirming the circular structure of circAβ-a. Sanger sequencing of the corresponding RT-PCR product confirmed the anticipated sequence of circAβ-a (Supplementary data-2). The resulting circAβ-a junction sequence is displayed in Figure 1C. In summary, we demonstrated the presence of circAβ-a in the human brain, both in AD and non-dementia samples by RT-PCR and sequencing.

### 3.2. Presence of the Additional APP hnRNA-Derived Circular RNAs in Human Brain

We examined whether additional *APP* primary transcript-derived circRNAs containing the Aβ coding exons (exon 17) could be detected. To address this question experimentally, we designed divergent RT-PCR primer pairs that target exon 17 of *APP* (Supplementary data-3). Total RNA isolated from human prefrontal lobe and hippocampus was used as a template for RT-PCR amplification. Analysis of the resulting amplicons by 5% native polyacrylamide gel electrophoresis revealed a number of distinct signals (Supplementary data-3). Deep sequencing of the resulting RT-PCR products identified 17 circRNAs derived from the *APP* gene with circAβ-a being the most abundant. Importantly, RNase R treatment confirmed their circular structure (Supplementary data-3). In addition to circAβ-a, we detected 16 circRNA isoforms harboring exon 17 of the *APP* gene. The resulting variants were systematically designated as circAβ-b, c,...q. Finally, we used sets of unique divergent primer pairs to verify the expression of circAβ-b and circAβ-c in the human brain of three AD patients and three non-dementia control samples (prefrontal cortex) by RT-PCR (Supplementary data-3). These data provided a survey of circRNA biogenesis from the Aβ encoding exons of the *APP* gene. The most recent update of circRNA database (circAltas) lists several additional circRNA candidates from other exons of the *APP* gene primary transcript [67].

### 3.3. Intron-Mediated Enhancement (IME) Promotes circAβ-a Overexpression in Human Cells

We recently established a novel system for the analysis of putative circRNA functions based on intron mediated enhancement [57]. This approach enables the efficient expression of circRNAs in transient transfection assays and the investigation of circRNA translation mechanisms. We adapted this universal method to analyze the putative protein coding potential of circAβ-a, as it is the most abundant copy of the 17 detected circRNA variants (Supplementary data-3). For this purpose, a cDNA representing APP protein coding exons 14–17 was inserted into pCircRNA-DMo plasmid, which harbors a chimeric intron to enhance circRNA formation (Figure 1D) [57]. In transiently transfected HEK293 cells, the resulting construct (pCircRNA-DMo-Aβ-a) demonstrated robust circAβ-a levels (Figure 1E). Importantly, agarose gel electrophoresis of RT-PCR products confirmed that the product of heterologous circAβ-a formation in HEK293 cells and the endogenous transcript in the human brain are of identical size (Figure 1A,E). Analysis of the total RNA by qRT-PCR revealed ~3000-fold overexpression of circAβ-a, compared to the endogenous background levels in HEK293 cells (Figure 1F).

For further validation, we treated total RNA with RNase R prior to Northern blot hybridization. This analysis required controls for RNase R digestion and Northern hybridization. For this purpose, we generated vector pCMV-Aβ175-cDNA, which enabled the expression of the linear circAβ-a RNA variant in control transfections. Notably, this construct also included the C-terminal non-canonical amino acid residues that were unique to the circular template. Analysis of transfection assays by Northern blot hybridization revealed two specific signals of different sizes in 5% native polyacrylamide gels, representing the circRNA and its linear mRNA counterpart (Figure 1G). The slower migrating RNA in pCMV-Aβ175-cDNA transfections was not detectable after RNase R treatment (Figure 1G), suggesting that this signal corresponds to the linear variant of circAβ-a. The faster migrating RNA in circAβ-a transfections (pCircRNA-DMo-Aβ-a) was resistant to RNase R digestion, in agreement with its circular structure (Figure 1G). Similar to our previous report [57], no linear RNA variants were detected by Northern blot hybridizations in pCircRNA-DMo-Aβ-a transfections, minimizing chances of linear RNA ‘contamination’ in subsequent assays (see below).

By RT-PCR amplification and subsequent Sanger sequencing with a third pair of oligonucleotide primers (circAβ-a_F3 and circAβ-a_R3, Figure 1A), we reconfirmed the correct splice junction of circAβ-a in HEK 293 cell transfection experiments (Figure 1H,I). In addition, the qRT-qPCR analysis of APP mRNA revealed largely unaffected endogenous APP mRNA levels (Supplementary data-4). We concluded that our IME strategy enabled strong circAβ-a formation in HEK293 cells. Notably, HEK293 cells represent a well-established cellular model for Alzheimer’s disease [61,62]; our system is therefore appropriate for the ex vivo analysis of potential circAβ-a functions in AD.

### 3.4. circAβ-a can be Translated into an Aβ-Related Protein in Human Cells

**Figure 2:**
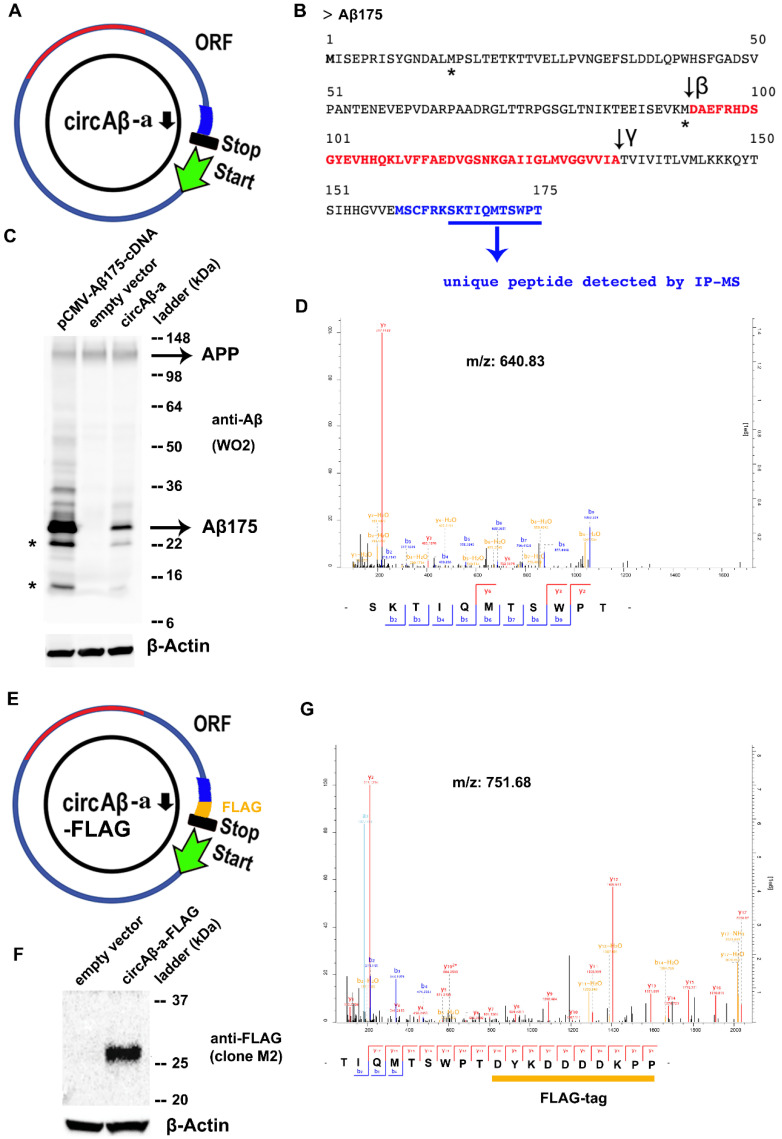
Analysis of circAβ-a translation and Aβ-related protein (Aβ175) in HEK293 cells. (**A**). The open reading frame (ORF) of circAβ-a is represented by a blue circle. The wider blue segment depicts the unique peptide sequence of the circAβ-a translated protein, the green arrow indicates the presumed translation start codon, the black bar represents the translational stop codon; the inner black arrow indicates the 5′ nucleotide of exon 14. (**B**). The predicted polypeptide sequence of Aβ175. Black and red letters represent the polypeptide sequence of Aβ175, which is identical to the corresponding segment of wild type APP protein. Processing sites for β and γ secretases are indicated by black arrows, the Aβ42 amino acid sequence is depicted by red letters, blue letters indicate the unique C-terminal sequence of Aβ175 (which is not present in APP full-length protein), blue underlined letters indicate the amino acids of the unique peptide detected by IP-MS as displayed in (**D**). (**C**). Western blot analysis of Aβ175 in HEK293 cells. pCMV-Aβ175-cDNA indicates linear, i.e., canonical mRNA-driven expression of Aβ175 cDNA. Mock transfection with empty vector (pCircRNA-DMo) was included as control. circAβ-a indicates expression after pCircRNA-DMo-Aβ-a transfections in HEK293 cells. The peptides detected are indicated on the right, Aβ175 marks the migration of the circAβ-a-derived protein, β-actin served as the loading control. SeeBlue™ Plus2 pre-stained protein standard was utilized as a protein size marker. * represent possible protein products that were the result of alternative translational initiation at AUG codons further downstream or processing products. (**D**). Mass spectrometry to demonstrate circular circAβ-a translation. The polypeptide harboring non-APP sequences (see B for details) was enriched via immunoprecipitation of Aβ175 with the anti-Aβ antibody in HEK293 cells transfected with the pCircRNA-DMo-Aβ-a expression vector (4G8 and 6E10), the scale of the left ordinate is the relative signal intensity, absolute intensities are displayed by the ordinate to the right, the *x*-axis denotes m/z values. (**E**). FLAG-tagged circAβ-a (circAβ-a-FLAG). The FLAG peptide tag, i.e., DYKDDDDKPP, was fused to the C-terminus of the circAβ-a ORF (yellow). (**F**). Western blot analysis for Aβ175-FLAG expression in N2a cells with anti-FLAG antibody (M2). Control represents empty vector (pCircRNA-DMo) mock transfections, circAβ-a-FLAG indicates expression of the pCircRNA-DMo-Aβ-a-FLAG vector, Precision plus protein™ dual color Standards served as size marker and β-actin as the loading control. (**G**). Mass spectrometry of the unique peptide representing a circular translation of circAβ-a-FLAG. This peptide was enriched via immunoprecipitation of Aβ175 with anti-Aβ antibody (4G8 and 6E10), the scale of the left ordinate is the relative signal intensity, absolute intensities are displayed by the right ordinate, the *x*-axis denotes m/z values.

Recent publications report that circRNA open reading frames (ORFs) can act as templates for efficient protein biosynthesis [57,58,60]. We identified a hypothetical 528 nt long ORF, presumably beginning at the first potential in-frame AUG start codon in exon 14 and ending, after what corresponds to the 3′ end of exon 17. The latter exon continues into exon 14, due to the circular nature of the RNA template. Because the 524 nt of the circle is not divisible by three, translation continues until it encounters, after 17 additional codons out-of-frame, a stop codon in exon 14. Consequently, the C-terminus of the circAβ-a sequence (maximally 175 amino acids with a predicted MW of 19.2 kDa, see Figure 2, Supplementary data-5) was different from any aa sequence translated from linear APP mRNA. Circumstantial evidence points to AUG (nucleotide position 51–53, amino acid position 1 of the sequence, see Supplementary data-5) as the start codon. The first two AUG codons in the sequence are out-of-frame and, if used, could not lead to the peptides detected by IP-MS (see below). The fourth potential AUG start codon can be ruled out, due to peptide 1 detected by IP-MS (see below). More distally, in-frame AUG codons (the next one is located adjacent to the Aβ42 peptide) can also be excluded, because of the size of the polypeptide (in the predicted to observed range of ~19–25 kDa (Figure 2B,C,E). We performed a search for internal ribosome entry sites (IRES) using IRESbase [65] and failed to identify a match; however, an A/U-rich sequence was detected upstream from the assumed start codon at nucleotide position 51–53 (Supplementary data-5). Recently, Fan et al. reported that AU-rich sequences also have the potential to serve as IRES-like elements in diverse circRNAs [68](preprint). This circAβ-a-derived protein is henceforth referred to as Aβ175 (Figure 2B, see below). To investigate whether circAβ-a encodes a bona fide protein, we evaluated Aβ175 expression in transient transfection assays of pCircRNA-DMo-Aβ-a in HEK293 cells by Western blot analysis. In order to avoid proteolytic processing of Aβ175, several secretase inhibitors were added to the cell culture medium (Materials and Methods). Plasmid pCMV-Aβ175-cDNA, expressing the linear mRNA form of circAβ-a ORF (see above), was utilized in parallel transfection assays and served as positive control. Western blot analysis with the amyloid beta peptide antibody (WO2) revealed that the circAβ-a ORF is efficiently translated into an Aβ-related protein of apparently 25 kDa (migrating between the 22 kDa and 36 kDa size markers) (Figure 2C). Notably, signals for the circAβ-a-related protein and the positive control (pCMV-Aβ175-cDNA) were of identical size.

The translation product of circAβ-a was not entirely identical to the corresponding protein segment of APP, due to the unique 17 amino acid (aa) extension at the C-terminus of Aβ175 protein. The presence of these aa residues is the result of circular translation into a different open reading frame at the splice junction of exon 17 to 14 (see above). Therefore, a part of the APP CDS exon 14 is translated out-of-frame compared to the original full-length (linear) APP, where this polypeptide domain is entirely absent (Figure 2A,B, Supplementary data-5). To provide further experimental evidence for circAβ-a translation, we performed mass spectrometry analysis to verify Aβ175 specific peptides. For enrichment, Aβ175 was immunoprecipitated with anti-Aβ antibodies (6E10, 4G8) from lysates of circAβ-a overexpressing HEK293 cells. Subsequent mass spectrometry revealed a peptide (SKTIQMTSWPT) corresponding to a part of the unique C-terminal portion of Aβ175 (Figure 2B,D). Therefore, this analysis provided strong evidence for circAβ-a translation in the HEK293 transfection assays. In order to discriminate the circRNA encoded polypeptide from similar endogenous products, we modified the C-terminal portion of Aβ175 in the circAβ-a ORF adding the FLAG-tag sequence (DYKDDDDKPP). The resulting recombinant expression vector was designated as pCircRNA-DMo-Aβ-a-FLAG (Figure 2E). To cross validate circAβ-a-FLAG translation in a more relevant neuronal cell line, we utilized transient transfection assays in mouse neuroblastoma N2a cells (N2a) cells, which enabled particularly efficient transfections. Western blot analysis with an anti-FLAG monoclonal antibody (M2) revealed a specific signal of approximately 25 kDa (Figure 2F), confirming that circAβ-a-FLAG was also an efficient template for protein biosynthesis in N2a cells. Similar to our HEK 293 cell system (see above) we used mass spectrometry to further validate the Western blot results. The FLAG-tagged protein (Aβ175-FLAG) was enriched via immunoprecipitation with anti-Aβ antibodies (6E10, 4G8) in circAβ-a-FLAG overexpressing N2a cells. Subsequent mass spectrometry identified a peptide specific to circAβ-a-FLAG translation; in fact, it represented the C-terminal fusion of Aβ175 and the added FLAG tag (TIQMTSWPTDYKDDDDKPP, Figure 2G). This finding corroborated translation of circAβ-a-FLAG in living cells.

### 3.5. Expression of circAβ-a Translated Protein (Aβ175) in Human Brain

**Figure 3:**
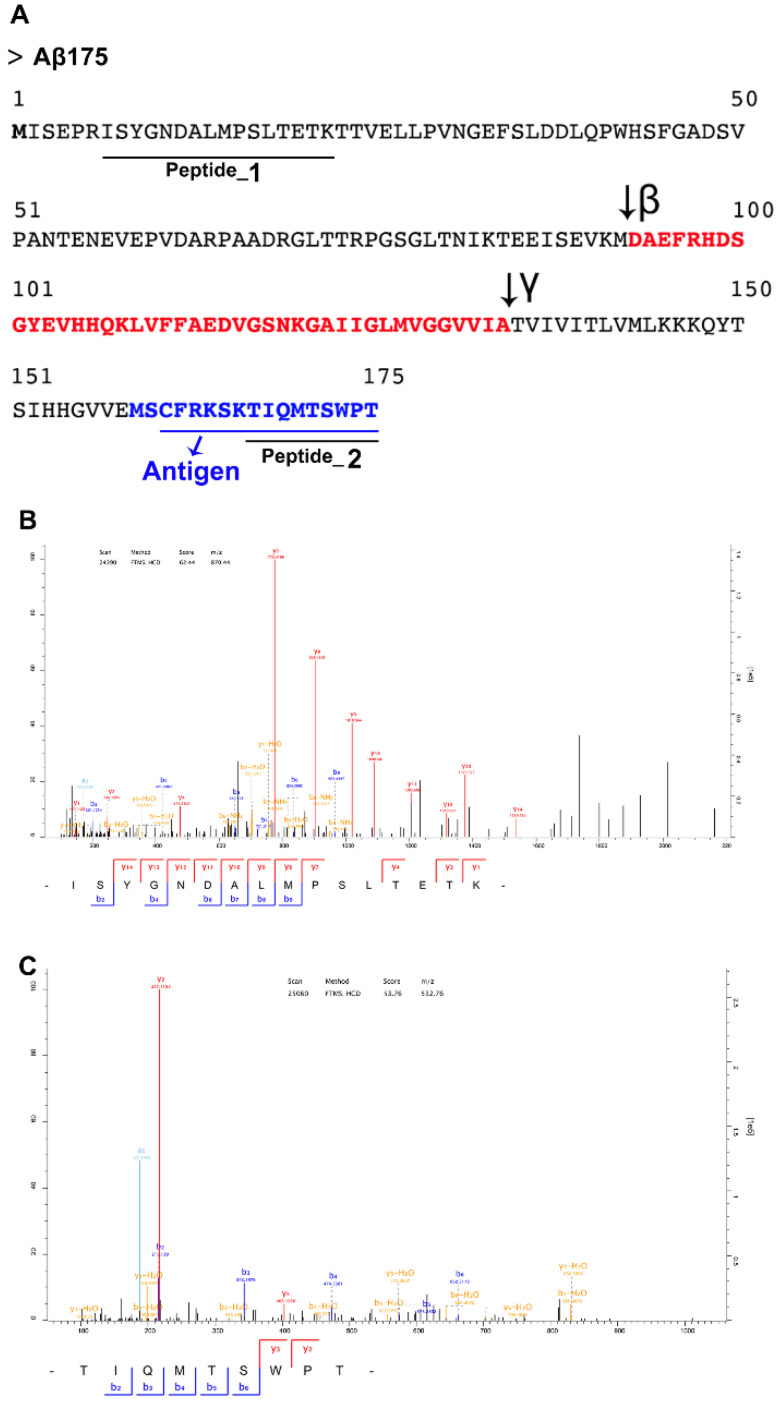
Immunoprecipitation–mass spectrometry (IP-MS) confirms the expression of Aβ175 in human brain. (**A**). Antigen and peptides identified by IP-MS are mapped to the predicted polypeptide sequence of Aβ175. The sequence underlined in blue denotes the antigen utilized for rabbit polyclonal antibody production (anti-Aβ175). Peptide sequences underlined in black indicate peptides detected by IP-MS: peptide_1, detected peptide shown in (**B**); peptide_2, detected peptide shown in (**C**); (**B**,**C**) mass spectrometry of detected peptides by IP-MS in human prefrontal cortex with anti-Aβ175. We searched the human genome by UniProt database to determine whether, theoretically, this peptide could be generated from another locus different than the APP gene, and found that is it unique to circAβ-a.

In cell lines, we demonstrated that circAβ-a was efficiently translated into an Aβ-containing polypeptide. These data might imply functional roles of these circRNAs and their polypeptide products in the pathology of AD. For in vivo analysis of a potential circAβ-a related role in human brain, we screened samples for Aβ175 (circAβ-a derived protein). A rabbit polyclonal antibody (anti-Aβ175) directed against the C-terminal domain of Aβ175 was generated (Figure 3A). As shown above, the C-terminus of Aβ175 harbored 17 additional unique amino acids. We assumed that an antibody raised against this antigenic domain would distinguish Aβ175 from products derived from full length APP translated from linear mRNA; however, Western blot analysis of human brain samples using anti-Aβ175 antibody revealed low specificity (Supplementary data-6). Nevertheless, our analysis identified, among other signals, one in the predicted size range, presumably corresponding to Aβ175 protein, which was also expressed in HEK293 cells (positive control). To confirm the identity of the putative Aβ175 protein detected in the Western blot, we performed immune precipitation of human brain extracts (in RIPA buffer) with anti-Aβ175 antibody followed by mass spectrometry (IP-MS) analysis of the precipitates. We identified two peptides: One located close to the predicted Aβ175 N-terminus (peptide_1 in Figure 3A,B, Supplementary data-5) and the second mapped to the C-terminal region, covering the unique (non-APP related) portion of Aβ175 (peptide_2 in Figure 3A,C, Supplementary data-5). These results were consistent with the in silico determined amino acid sequence (Figure 3A, Supplementary data-5) as well as our ex vivo analysis (see above). Our data demonstrated that circAβ-a was translated into Aβ-related protein (Aβ175) in human brain, thereby representing an additional source of Aβ peptides in vivo.

### 3.6. Aβ175 Generates Aβ Peptides in HEK293 Cells

**Figure 4:**
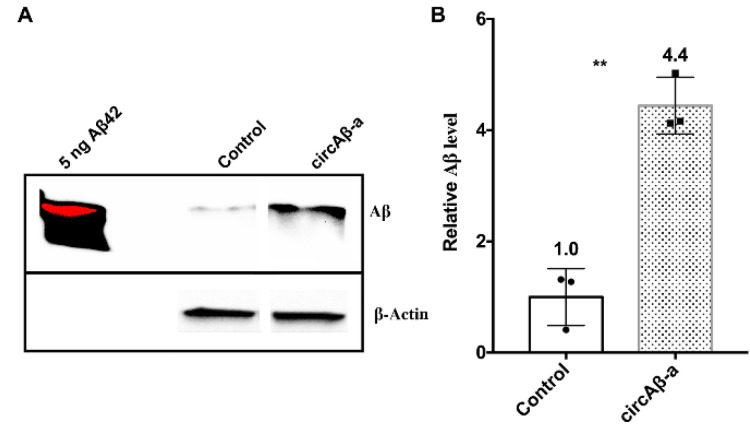
circAβ-a overexpression generates Aβ peptides. (**A**). Immunoprecipitation–Western blotting (IP-WB) of Aβ peptides in the conditioned medium of circAβ-a overexpressing cells. Conditioned cell culture medium for HEK293 cells, transfected with the circAβ-a overexpression vector, was utilized for immunoprecipitation with antibodies against Aβ (6E10, 4G8; mouse antibodies). Control represents the IP-WB results for mock transfections (pCircRNA-DMo), circAβ-a indicates pCircRNA-DMo-Aβ-a transfections, rabbit D54D2 antibody specific for Aβ was utilized in this Western blot analysis, β-actin served as loading control and 5 ng of in vitro synthesized Aβ42 served as Aβ migration maker. The red color for the Aβ42 signal in the left lane was the result of over-exposure. (**B**). Quantification of A. Student’s T-tests were performed against the control sample; **, *p* ≤ 0.01; *n* = 3. Details for the three replicates of IP-WB results are provided in Supplementary data-7.

Translation of Aβ-related protein from circAβ-a RNA templates in our cell culture assays and human brain suggested a putative role of circAβ-a in the biogenesis of Aβ peptides. The amino acid sequence of Aβ175 contains β and γ-secretase cleavage sites. This implies that amyloid beta peptides might be generated from Aβ175 via β- and γ-secretase-mediated cleavage (Figure 2B). For circAβ-a overexpressing HEK293 cells, we utilized immunoprecipitation coupled with Western blotting (IP-WB) using anti-Aβ-antibodies (for IP: 6E10, 4G8, mouse antibody) to identify Aβ peptides in conditioned cell culture medium (CM). We obtained a specific signal corresponding to Aβ by Western blot analysis (i.e., after IP) with the Aβ antibody D54D2 (rabbit antibody). Compared to HEK293 mock transfections with empty vector, which served as negative control, pCircRNA-DMo-Aβ-a enhanced the expression of the Aβ peptide by more than 4.4-fold (Figure 4A,B, Supplementary data-7). These results confirmed an alternative route to Aβ production via circAβ-a and its translational product Aβ175 (Figure 4A,B).

## 4. Discussion

**Figure 5:**
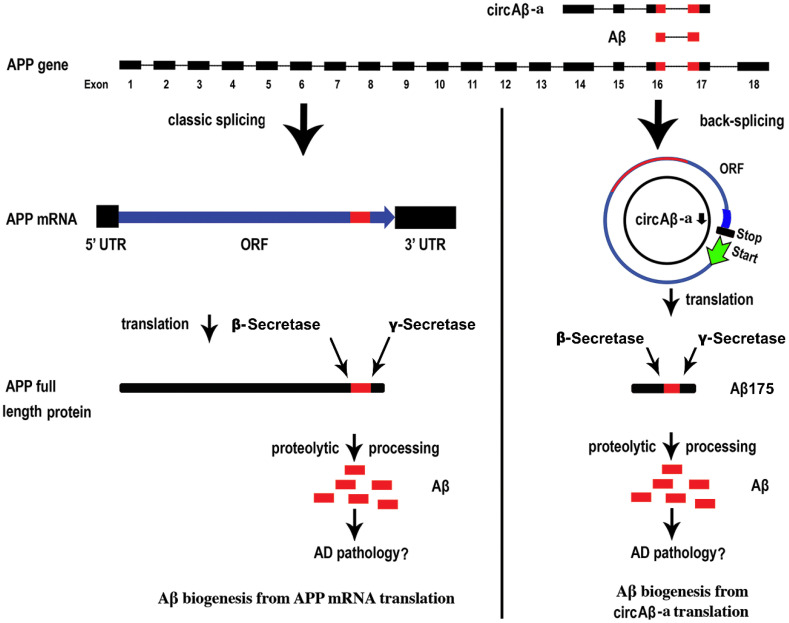
The alternative pathway of Aβ biogenesis in Alzheimer’s disease. At the top, exon sequences containing circAβ-a and Aβ peptides (in red) are aligned with the full-length *APP* gene (not drawn to scale). On the left, linear APP mRNA transcribed from the *APP* gene locus undergoes the canonical splicing pathway before being translated into full-length APP protein. Proteolytic processing of APP protein generates Aβ peptides (e.g., Aβ40, Aβ42, in red color), which may play causative roles in the AD pathology. On the right, circAβ-a is synthesized by back-splicing of the *APP* gene. The open reading frame (ORF) is in blue, with the Aβ sequence in red. The presumed translational start codon is depicted by a green arrow and the stop codon by a black bar. Translation of circAβ-a produces Aβ-related peptide (Aβ175), which is further processed to form Aβ peptides.

Although the biogenesis of Aβ in familial AD is well-documented, Aβ production in sporadic AD is not well understood [1,40,41,42]. We identified circAβ-a and analyzed its formation in prefrontal cortex samples of sporadic AD patients, non-dementia controls, and transient transfection assays. According to circbase, circAβ-a (hsa_circ_0007556) can be found in various human brain tissues (diencephalon, cerebellum, occipital lobe, frontal cortex, parietal lobe, temporal lobe) [63]. We also identified 16 additional circAβ forms both in frontal lobe and hippocampus. With the aid of intron-mediated enhancement [57], we confirmed that circAβ-a served as a template for the biosynthesis of an Aβ-related protein (Aβ175) in HEK293 cells. In addition, the unique C-terminal peptide of Aβ175 was identified in human brain samples. Furthermore, Aβ175 was processed into Aβ peptides, representing a novel route of Aβ generation that might give rise to new perspectives on the molecular mechanisms leading to the manifestation of Alzheimer’s disease (Figure 5). This mechanism is substantially different from the canonical Aβ accumulation caused by the pathophysiological dysregulation of full-length APP proteolytic processing (Figure 5) [14]. In particular, unlike specific mutations in the *APP* gene, which are commonly known to be responsible for familial forms of AD [14], no such specific mutations are required for the biogenesis of circAβ-a. circRNA biogenesis is largely directed by the complementary elements in surrounding intronic regions [48]. It is likely that all human individuals generate circAβ-a RNA, indicating a possible role in the pathogenesis of sporadic AD. It has already been demonstrated that translation of some circRNA could be activated under certain stress conditions [58,69,70]. Further analysis is required to investigate whether circAβ-a level and its translation is elevated in the aging brain (especially in AD pathology), which could be triggered by endogenous or environmental conditions eliciting various cellular stresses, such as oxidative damage [71,72,73].

We performed a search for internal ribosome entry sites (IRES) using the IRESbase database [65] and no matches were detected. This suggested that the definition of IRES criteria used in the database may have to be broadened for circRNAs. Recently, specific criteria of IRES prediction in circRNAs were investigated by Fan et al. [68] (preprint). Accordingly, we identified a putative AU-rich element near the predicted start codon of circAβ-a, which might activate cap-independent translation in circAβ-a (Supplementary data-5).

Apart from the circAβ-a studied here, it will be interesting to delineate the possible roles of the additional 16 circAβ isoforms, for example whether their abundance changes in AD pathology and whether these isoforms could also be potential RNA templates for Aβ related polypeptides.

It has recently been suggested that ciRS-7 could play a role in Alzheimer’s disease (AD) by targeting miRNA-7, which has been shown to play regulatory roles in the AD pathology [74,75]. Here, we focused on the direct production of Aβ peptides by circAβ isoforms, but we did not exclude possible indirect regulatory functions of circAβ isoforms such as regulation miRNA/mRNA interactions [76].

Recently, circRNA expression and AD pathology correlation was investigated. With extensive RNA-sequencing of AD brain samples, Dube et al. report that 10 circRNAs have strong pathological associations with AD [55]. Quite surprisingly, circAβ-a and its other isoforms were not included. This could be due to insufficient depth of sequencing with respect to circAβ isoforms as a result of bias in library construction [77]. Furthermore, the involvement of circAβ variants in AD pathology may lie in their translation activation, rather than changes in their circRNA levels. Interestingly, the mouse orthologue of circRTN4, one of the circRNAs with strong correlation to AD, has been previously reported to produce proteins (both monomer and repeating multimers by rolling cycle translation) [57]. Meanwhile, RTN4 protein (NogoA) is known to play a role in AD through BACE1 activity regulation [78]. Thus, circRTN4 derived polypeptides may have a regulatory role in AD akin to its NogoA protein counterpart. Additional polypeptide-producing circRNAs with strong AD associations could possibly play a role. In any event, the investigation of biological roles of circAβ-a offers new perspectives in the search for underlying mechanisms of sporadic Alzheimer’s disease, which could ultimately lead to the design of disease-modifying drugs [56].

Future studies should examine the ratio of circAβ-a translated Aβ peptides compared to those derived from APP full-length protein. Furthermore, compared to full-length APP protein, Aβ175 lacks a signal peptide (17aa), as well as 487 aa of the N-terminal portions, has a shorter and modified C-terminus and presumably a different tertiary structure. Consequently, it will be worthwhile to determine whether a different structure exposes the amino acid residues relevant for Aβ175 secretase processing, thus generating more Aβ peptides; this is of particular significance in the context of the aging human brain and neurodegenerative diseases.

Importantly, we confirmed translation of circular RNA into polypeptide variants of canonical proteins in cultured cells or human tissue. Alternative splicing of primary transcripts has long been appreciated as one way to generate multiple polypeptide variants out of a single gene [79,80,81]. This includes recruitment of novel exons out of intronic space [82,83,84,85]. Circular RNA can generate further polypeptide variants, such as multimers of the respective exonic domains, provided the circRNA template does not contain a stop codon in frame and is divisible by three [57] or, as shown here, adds novel polypeptide sequences after initial in-frame translation of the circle continues out-of-frame [86], thus increasing proteome complexity. Albeit many, if not most, circular RNAs are probably neutral or slightly detrimental and non-functional by-products of hnRNA processing, a few might have detrimental effects for the organism by triggering cellular processes leading to disease; this might be the case for some of the circRNAs presented here. On the other hand, a few circRNAs, whether translated or not, might constitute an exaptation when the RNA or its translation product was co-opted into a variant or novel cellular function [85,87,88].

## Supplementary Materials

The following are available online at https://www.mdpi.com/2073-4409/9/10/2196/s1, Supplementary data-1, 2, 3, 4, 5, 6, 7, Table S1.
