# Supplementary material for: Circular RNA Encoded Amyloid Beta peptides—A Novel Putative Player in Alzheimer’s Disease
Source: Cells. 2020 Sep 29;9(10):2196. doi: 10.3390/cells9102196 (PMC7650678; doi:10.3390/cells9102196)
Supplement: Supplementary file 1 [file cells-09-02196-s001.zip › revised supplementary data/Supplementary Table 1-final.docx]

**Supplementary Table-1**

DNA oligonucleotides used in this study

| human circAβ-a expression  plasmid construction oligos | Oligonucleotide sequences: from 5’ to 3’ |
| --- | --- |
| Aβ-VF2 | GTGATCGTCATCACCTTGGTGATGC |
| Aβ-VR2 | CACCATGAGTCCAATGATTGCACC |
| Abeta-circF | GTTTGTTTTTCAGATGAGCTGCTTCAGAAAGAGCAAAACT |
| ABeta-circR | GCATGGATTATTACCTCCACCACACCATGATGAATGG |
| circDMO-LF | GTCGACTGGATCCAACGTTAACCC |
| DMo-Ab-LR | CTGAAGCAGCTCATCTGAAAAACAAACAGAATACAACCTCAGC |
| DMo-Ab-RF | GGTGTGGTGGAGGTAATAATCCATGCACCGTCTCACC |
| circDMO-RR | CACTTTGCTCGAGCTCATCAACATG |
| oligonucleotides for human  circAβ-a identification |  |
| circAβ-a-R1 | GAAGCAGCTCATCTCCACCACACC |
| circAβ-a-F1 | CGTCTTGGCCAACATGATTAGTGAACC |
| circAβ-a-F2 | GTCATAGCGACAGTGATCGTC |
| circAβ-a-R2 | CTTGGTTCACTAATCATGTTGGC |
| circAβ-a-F3 | GTGATCGTCATCACCTTGGTGATGC |
| circAβ-a-R3 | CACCATGAGTCCAATGATTGCACC |
| oligonucleotides for human  APP mRNA qRT-PCR |  |
| hAPP-mF | TTTGTGATTCCCTACCGCTG |
| hAPP-mR | TGCCAGTGAAGATGAGTTTCG |
| Human ACTB mRNA: |  |
| hACTB-F | ACCTTCTACAATGAGCTGCG |
| hACTB-R | CCTGGATAGCAACGTACATGG |
