# Supplementary material for: Circular RNA Encoded Amyloid Beta peptides—A Novel Putative Player in Alzheimer’s Disease
Source: Cells. 2020 Sep 29;9(10):2196. doi: 10.3390/cells9102196 (PMC7650678; doi:10.3390/cells9102196)
Supplement: Supplementary file 1 [file cells-09-02196-s001.zip › revised supplementary data/Supplementary data-7_final.docx]

**Supplementary data-7**


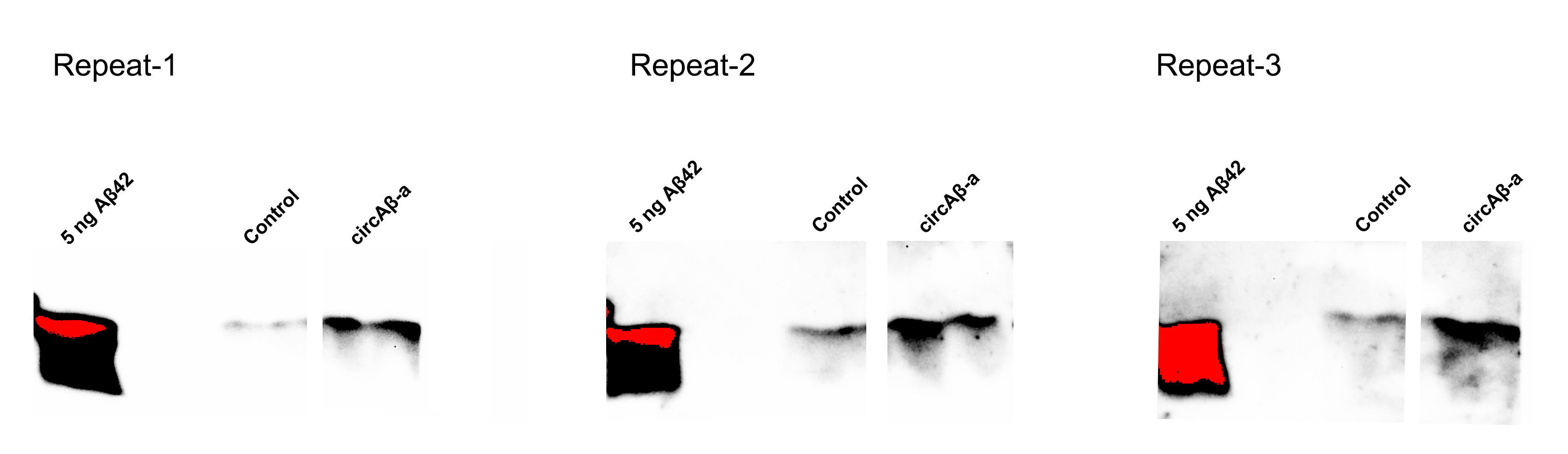


**circAβ-a overexpression generates Aβ peptides.**

**Immunoprecipitation/Western blotting (IP-WB) of Aβ peptides in the conditioned medium of circAβ-a overexpressing cells**. Conditioned cell culture medium for HEK293 cells, transfected with the circAβ-a overexpression vector was utilized for immunoprecipitation with antibodies against Aβ (6E10, 4G8; mouse antibodies). Control represents the IP-WB results for mock transfections (pCircRNA-DMo), circAβ-a indicates pCircRNA-DMo-Aβ-a transfections, rabbit D54D2 antibody specific for Aβ was utilized in this Western blot analysis, β-Actin served as loading control and 5 ng of *in vitro* synthesized Aβ42 were added as Aβ migration maker. The red color for the Aβ42 signal in the left most lane was the result of over-exposure. Three repeats are presented here.
