# Supplementary material for: Circular RNA Encoded Amyloid Beta peptides—A Novel Putative Player in Alzheimer’s Disease
Source: Cells. 2020 Sep 29;9(10):2196. doi: 10.3390/cells9102196 (PMC7650678; doi:10.3390/cells9102196)
Supplement: Supplementary file 1 [file cells-09-02196-s001.zip › revised supplementary data/Supplementary data-2-final.pdf]

Project: Untitled.sqd Contig 1

```
                                                        10        20        30        40
                                                ----:----|----:----|----:----|----:----|----
                                                TTGTCATAGCGACAGTGATCGTCATCACCTTGGTGATGCTGAAG

AD3-R-60_1.seq(7>104)                       ←   TTGTCATAGCGACAGTGATCGTCATCACCTTGGTGATGCTGAAG
ND3-R-61_1.seq(4>98)                        ←      TCATAGCGACAGTGATCGTCATCACCTTGGTGATGCTGAAG
circAB-a-junction  region-WT.seq(1>148)     →     GTCATAGCGACAGTGATCGTCATCACCTTGGTGATGCTGAAG

                                                     50        60        70        80
                                                ----:----|----:----|----:----|----:----|----
                                                AAGAAACAGTACACATCCATTCATCATGGTGTGGTGGAGATGAG

AD3-R-60_1.seq(7>104)                       ←   AAGAAACAGTACACATCCATTCATCATGGTGTGGTGGAGATGAG
ND3-R-61_1.seq(4>98)                        ←   AAGAAACAGTACACATCCATTCATCATGGTGTGGTGGAGATGAG
circAB-a-junction  region-WT.seq(1>148)     →   AAGAAACAGTACACATCCATTCATCATGGTGTGGTGGAGATGAG
ND3-F-52_1.seq(14>113)                      →           GTACACATCCATTCATCATGGTGTGGTGGAGATGAG
AD3-F-51_1.seq(3>107)                       →           GTACACATCCATTCATCATGGTGTGGTGGAGATGAG

                                                90        100       110       120       130
                                                ----:----|----:----|----:----|----:----|----
                                                CTGCTTCAGAAAGAGCAAAACTATTCAGATGACGTCTTGGCCAA

AD3-R-60_1.seq(7>104)                       ←   CTGCTTCAGA
ND3-R-61_1.seq(4>98)                        ←   CTGCTTCAGA
circAB-a-junction  region-WT.seq(1>148)     →   CTGCTTCAGAAAGAGCAAAACTATTCAGATGACGTCTTGGCCAA
ND3-F-52_1.seq(14>113)                      →   CTGCTTCAGAAAGAGCAAAACTATTCAGATGACGTCTTGGCCAA
AD3-F-51_1.seq(3>107)                       →   CTGCTTCAGAAAGAGCAAAACTATTCAGATGACGTCTTGGCCAA

                                                      140       150
                                                ----:----|----:----|----:
                                                CATGATTAGTGAACCAAGAAGCTTG

circAB-a-junction  region-WT.seq(1>148)     →   CATGATTAGTGAACCAAG
ND3-F-52_1.seq(14>113)                      →   CATGATTAGTGAACCAAGAA
AD3-F-51_1.seq(3>107)                       →   CATGATTAGTGAACCAAGAAGCTTG
```
