# Supplementary material for: Circular RNA Encoded Amyloid Beta peptides—A Novel Putative Player in Alzheimer’s Disease
Source: Cells. 2020 Sep 29;9(10):2196. doi: 10.3390/cells9102196 (PMC7650678; doi:10.3390/cells9102196)
Supplement: Supplementary file 1 [file cells-09-02196-s001.zip › revised supplementary data/Supplementary data-5-final.docx]

**Supplementary data-5**

**A. Open reading frame of circAβ-a**

5' end of exon 14

AUGAGCUGCUUCAGAAAGAGCAAAACUAUUCAGAUGACGUCUUGGCCAAC

AU-rich (IRES?)

**AUG**AUUAGUGAACCAAGGAUCAGUUACGGAAACGAUGCUCUCAUGCCAUC

**-M--**I**--**S**--**E**--**P**--**R**--**I**--**S**--**Y**--**G**--**N**--**D**--**A**--**L**--**M**--**P**--**S

1 17

UUUGACCGAAACGAAAACCACCGUGGAGCUCCUUCCCGUGAAUGGAGAGU

**--**L**--**T**--**E**--**T**--**K**--**T**--**T**--**V**--**E**--**L**--**L**--**P**--**V**--**N**--**G**--**E

18 33

UCAGCCUGGACGAUCUCCAGCCGUGGCAUUCUUUUGGGGCUGACUCUGUG

F**--**S**--**L**--**D**--**D**--**L**--**Q**--**P**--**W**--**H**--**S**--**F**--**G**--**A**--**D**--**S**--**V

34 50

5' end of exon 15

CCAGCCAACACAGAAAACGAAGUUGAGCCUGUUGAUGCCCGCCCUGCUGC

**-**P**--**A**--**N**--**T**--**E**--**N**--**E**--**V**--**E**--**P**--**V**--**D**--**A**--**R**--**P**--**A**--**A

51 67

5' end of exon 16

CGACCGAGGACUGACCACUCGACCAGGUUCUGGGUUGACAAAUAUCAAGA

**--**D**--**R**--**G**--**L**--**T**--**T**--**R**--**P**--**G**--**S**--**G**--**L**--**T**--**N**--**I**--**K**--**

68 83

CGGAGGAGAUCUCUGAAGUGAAGAUGGAUGCAGAAUUCCGACAUGACUCA

T**--**E**--**E**--**I**--**S**--**E**--**V**--**K**--**M**--D--A--E--F--R--H--D--S**

84 100

5' end of exon 17

GGAUAUGAAGUUCAUCAUCAAAAAUUGGUGUUCUUUGCAGAAGAUGUGGG

**-G--Y--E--V--H--H--Q--K--L--V--F--F--A--E--D--V--G**

101 117

UUCAAACAAAGGUGCAAUCAUUGGACUCAUGGUGGGCGGUGUUGUCAUAG

**-S--N--K--G--A--I--I--G--L--M--V--G--G--V--V--I--**

118 133

CGACAGUGAUCGUCAUCACCUUGGUGAUGCUGAAGAAGAAACAGUACACA

**A--**T**--**V**--**I**--**V**--**I**--**T**--**L**--**V**--**M**--**L**--**K**--**K**--**K**--**Q**--**Y**--**T

134 150

5' end of exon 14

UCCAUUCAUCAUGGUGUGGUGGAGAUGAGCUGCUUCAGAAAGAGCAAAAC

**-**S**--**I**--**H**--**H**--**G**--**V**--**V**--**E**--M--S--C--F--R--K--S--K--T**

151 167

UAUUCAGAUGACGUCUUGGCCAACA**UGA**UUAGUGAA...

**-I--Q--M--T--S--W--P--T**

168 175

**Translated sequence of circAβ-a.** Shown is slightly more than a full circle of circAβ-a (extended by the repeated relevant 5' portion of exon 14). For better identification of the regions corresponding to the exons, they are highlighted in an alternating fashion in grey (exons 15 and 17) or are left unmodified (exons 14 and 16). The putative AUG start codon and the corresponding methionine residue (M) is shown in bold green lettering and the stop codon in bold dark red. The Aβ42 amino acid sequence is shown in red lettering and the additional C-terminal amino acids of Aβ175 encoded by exon 14 out of frame, due to the circular nature of the RNA template, are shown in blue. These 17 specific amino acids cannot be generated by the canonical APP mRNA. Peptides confirmed by mass spectrometry are underlined here. A possible internal ribosomal entry site (IRES) consisting of an AU-rich element is underlined [1].

>Aβ175, putative sequence

MISEPRISYGNDALMPSLTETKTTVELLPVNGEFSLDDLQPWHSFGADSVPANTENEVEPVDARPAADRGLTTRPGSGLTNIKTEEISEVKM**DAEFRHDSGYEVHHQKLVFFAEDVGSNKGAIIGLMVGGVVIA**TVIVITLVMLKKKQYTSIHHGVVE**MSCFRKSKTIQMTSWPT**

The Aβ42 sequence is shown in red. The 17 additional C-terminal amino acids encoded by the circular RNA are shown in blue.

**B. Alignment of Aβ175 with APP695**


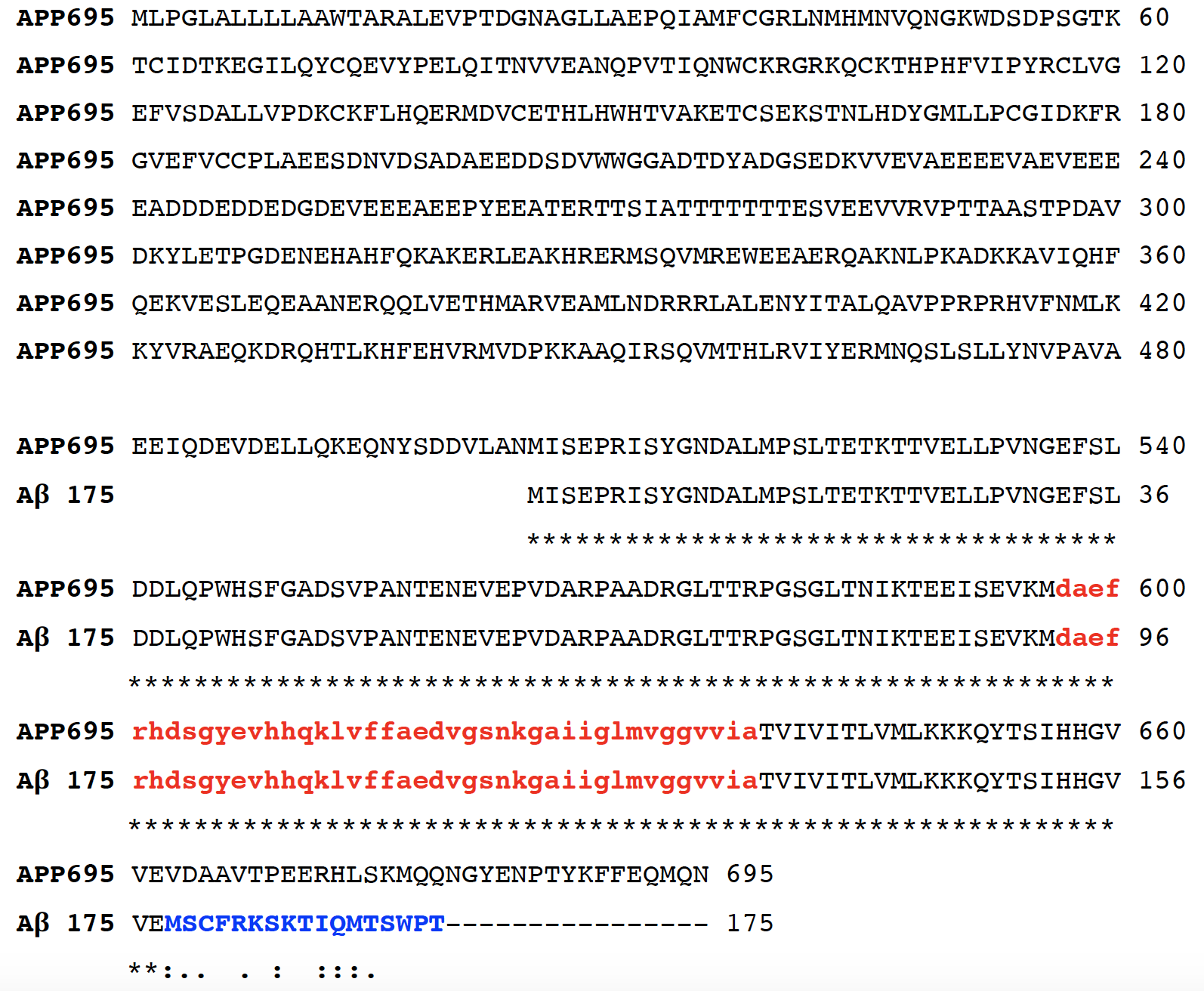


Protein sequence alignment between APP695 and Aβ175 was done through Clustal Omega (<https://www.ebi.ac.uk/Tools/msa/clustalo/>). Aβ42 sequence is shown in red color with lowercase.

Reference

1. Fan, X., et al., *Pervasive translation of circular RNAs driven by short IRES-like elements.* bioRxiv, 2020: p. 473207.
