# Supplementary material for: Circular RNA Encoded Amyloid Beta peptides—A Novel Putative Player in Alzheimer’s Disease
Source: Cells. 2020 Sep 29;9(10):2196. doi: 10.3390/cells9102196 (PMC7650678; doi:10.3390/cells9102196)
Supplement: Supplementary file 1 [file cells-09-02196-s001.zip › revised supplementary data/Supplementary data-1-final.pdf]

```
                                          10        20        30        40        50
                                   CGTCTTGG-CCAACATGATTAGTGAACCAAGGATCAGTTACGGAAACGAT

circAB-a-JR1-amplicon.seq(1>499)  → CGTCTTGG-CCAACATGATTAGTGAACCAAGGATCAGTTACGGAAACGAT
R-AD3-R-58_1.seq(8>437)           ←              CATGATTAGTGAACCAAGGATCAGTTACGGAAACGAT
R-ND3-R-59_1.seq(17>460)          ←             ACATGATTAGTGAACCAAGGATCAGTTACGGAAACGAT
AD1-R-48_1.seq(31>437)            ←             ACATGATTAGTGAACCAAGGATCAGTTACGGAAACGAT
R-ND3-F-50_1.seq(9>455)           →                                             AACGAT

                                          60        70        80        90       100
                                   GCTCTCATGC-CAT-CTTTGACCGAAACGAAAAC-CACCGTGGAGCTCCT

circAB-a-JR1-amplicon.seq(1>499)  → GCTCTCATGC-CAT-CTTTGACCGAAACGAAAAC-CACCGTGGAGCTCCT
R-AD3-R-58_1.seq(8>437)           ← GCTCTCATGC-CAT-CTTTGACCGAAACGAAAAC-CACCGTGGAGCTCCT
R-ND3-R-59_1.seq(17>460)          ← GCTCTCATGC-CAT-CTTTGACCGAAACGAAAAC-CACCGTGGAGCTCCT
AD1-R-48_1.seq(31>437)            ← GCTCTCATGC-CAT-CTTTGACCGAAACGAAAAC-CACCGTGGAGCTCCT
R-ND3-F-50_1.seq(9>455)           → GCTCTCATGC-CAT-CTTTGACCGAAACGAAAAC-CACCGTGGAGCTCCT
R-AD3-F-49_1.seq(9>440)           →            CAT-CTTTGACCGAAACGAAAAC-CACCGTGGAGCTCCT
AD1-F-45_1.seq(29>436)            →                                    CACCGTGGAGCTCCT

                                         110       120       130       140       150
                                   TCCCGTGAATGGAGAGTTCAGCCTGGACGATCTCCAGCCGTGGCATTCTT

circAB-a-JR1-amplicon.seq(1>499)  → TCCCGTGAATGGAGAGTTCAGCCTGGACGATCTCCAGCCGTGGCATTCTT
R-AD3-R-58_1.seq(8>437)           ← TCCCGTGAATGGAGAGTTCAGCCTGGACGATCTCCAGCCGTGGCATTCTT
R-ND3-R-59_1.seq(17>460)          ← TCCCGTGAATGGAGAGTTCAGCCTGGACGATCTCCAGCCGTGGCATTCTT
AD1-R-48_1.seq(31>437)            ← TCCCGTGAATGGAGAGTTCAGCCTGGACGATCTCCAGCCGTGGCATTCTT
R-ND3-F-50_1.seq(9>455)           → TCCCGTGAATGGAGAGTTCAGCCTGGACGATCTCCAGCCGTGGCATTCTT
R-AD3-F-49_1.seq(9>440)           → TCCCGTGAATGGAGAGTTCAGCCTGGACGATCTCCAGCCGTGGCATTCTT
AD1-F-45_1.seq(29>436)            → TCCCGTGAATGGAGAGTTCAGCCTGGACGATCTCCAGCCGTGGCATTCTT

                                         160       170       180       190       200
                                   TTGGGGCTGACTCTGTGCCAGCCAACACAGAAAACGAAGTTGAGCCTGTT

circAB-a-JR1-amplicon.seq(1>499)  → TTGGGGCTGACTCTGTGCCAGCCAACACAGAAAACGAAGTTGAGCCTGTT
R-AD3-R-58_1.seq(8>437)           ← TTGGGGCTGACTCTGTGCCAGCCAACACAGAAAACGAAGTTGAGCCTGTT
R-ND3-R-59_1.seq(17>460)          ← TTGGGGCTGACTCTGTGCCAGCCAACACAGAAAACGAAGTTGAGCCTGTT
AD1-R-48_1.seq(31>437)            ← TTGGGGCTGACTCTGTGCCAGCCAACACAGAAAACGAAGTTGAGCCTGTT
R-ND3-F-50_1.seq(9>455)           → TTGGGGCTGACTCTGTGCCAGCCAACACAGAAAACGAAGTTGAGCCTGTT
R-AD3-F-49_1.seq(9>440)           → TTGGGGCTGACTCTGTGCCAGCCAACACAGAAAACGAAGTTGAGCCTGTT
AD1-F-45_1.seq(29>436)            → TTGGGGCTGACTCTGTGCCAGCCAACACAGAAAACGAAGTTGAGCCTGTT

                                         210       220       230       240       250
                                   GATGCCCGCCCTGCTGCCGACCGAGGACTGACCACTCGACCAGGTTCTGG

circAB-a-JR1-amplicon.seq(1>499)  → GATGCCCGCCCTGCTGCCGACCGAGGACTGACCACTCGACCAGGTTCTGG
R-AD3-R-58_1.seq(8>437)           ← GATGCCCGCCCTGCTGCCGACCGAGGACTGACCACTCGACCAGGTTCTGG
R-ND3-R-59_1.seq(17>460)          ← GATGCCCGCCCTGCTGCCGACCGAGGACTGACCACTCGACCAGGTTCTGG
AD1-R-48_1.seq(31>437)            ← GATGCCCGCCCTGCTGCCGACCGAGGACTGACCACTCGACCAGGTTCTGG
R-ND3-F-50_1.seq(9>455)           → GATGCCCGCCCTGCTGCCGACCGAGGACTGACCACTCGACCAGGTTCTGG
R-AD3-F-49_1.seq(9>440)           → GATGCCCGCCCTGCTGCCGACCGAGGACTGACCACTCGACCAGGTTCTGG
AD1-F-45_1.seq(29>436)            → GATGCCCGCCCTGCTGCCGACCGAGGACTGACCACTCGACCAGGTTCTGG

                                         260       270       280       290       300
                                   GTTGACAAATATCAAGACGGAGGAGATCTCTGAAGTGAAGATGGATGCAG

circAB-a-JR1-amplicon.seq(1>499)  → GTTGACAAATATCAAGACGGAGGAGATCTCTGAAGTGAAGATGGATGCAG
R-AD3-R-58_1.seq(8>437)           ← GTTGACAAATATCAAGACGGAGGAGATCTCTGAAGTGAAGATGGATGCAG
R-ND3-R-59_1.seq(17>460)          ← GTTGACAAATATCAAGACGGAGGAGATCTCTGAAGTGAAGATGGATGCAG
AD1-R-48_1.seq(31>437)            ← GTTGACAAATATCAAGACGGAGGAGATCTCTGAAGTGAAGATGGATGCAG
R-ND3-F-50_1.seq(9>455)           → GTTGACAAATATCAAGACGGAGGAGATCTCTGAAGTGAAGATGGATGCAG
R-AD3-F-49_1.seq(9>440)           → GTTGACAAATATCAAGACGGAGGAGATCTCTGAAGTGAAGATGGATGCAG
AD1-F-45_1.seq(29>436)            → GTTGACAAATATCAAGACGGAGGAGATCTCTGAAGTGAAGATGGATGCAG
```

```
                                                    310       320       330       340       350
                                           AATTCCGACATGACTCAGGATATGAAGTTCATCATCAAAAATTGGTGTTC
circAB-a-JR1-amplicon.seq(1>499)      →    AATTCCGACATGACTCAGGATATGAAGTTCATCATCAAAAATTGGTGTTC
R-AD3-R-58_1.seq(8>437)               ←    AATTCCGACATGACTCAGGATATGAAGTTCATCATCAAAAATTGGTGTTC
R-ND3-R-59_1.seq(17>460)              ←    AATTCCGACATGACTCAGGATATGAAGTTCATCATCAAAAATTGGTGTTC
AD1-R-48_1.seq(31>437)                ←    AATTCCGACATGACTCAGGATATGAAGTTCATCATCAAAAATTGGTGTTC
R-ND3-F-50_1.seq(9>455)               →    AATTCCGACATGACTCAGGATATGAAGTTCATCATCAAAAATTGGTGTTC
R-AD3-F-49_1.seq(9>440)               →    AATTCCGACATGACTCAGGATATGAAGTTCATCATCAAAAATTGGTGTTC
AD1-F-45_1.seq(29>436)                →    AATTCCGACATGACTCAGGATATGAAGTTCATCATCAAAAATTGGTGTTC
```

```
                                                    360       370       380       390       400
                                           TTTGCAGAAGATGTGGGTTCAAACAAAGGTGCAATCATTGGACTCATGGT
circAB-a-JR1-amplicon.seq(1>499)      →    TTTGCAGAAGATGTGGGTTCAAACAAAGGTGCAATCATTGGACTCATGGT
R-AD3-R-58_1.seq(8>437)               ←    TTTGCAGAAGATGTGGGTTCAAACAAAGGTGCAATCATTGGACTCATGGT
R-ND3-R-59_1.seq(17>460)              ←    TTTGCAGAAGATGTGGGTTCAAACAAAGGTGCAATCATTGGACTCATGGT
AD1-R-48_1.seq(31>437)                ←    TTTGCAGAAGATGTGGGTTCAAACAAAGGTGCAATCATTGGACTCATGGT
R-ND3-F-50_1.seq(9>455)               →    TTTGCAGAAGATGTGGGTTCAAACAAAGGTGCAATCATTGGACTCATGGT
R-AD3-F-49_1.seq(9>440)               →    TTTGCAGAAGATGTGGGTTCAAACAAAGGTGCAATCATTGGACTCATGGT
AD1-F-45_1.seq(29>436)                →    TTTGCAGAAGATGTGGGTTCAAACAAAGGTGCAATCATTGGACTCATGGT
```

```
                                                    410       420       430       440       450
                                           GGGCGGTGTTGTCATAGCGACA-GTGATCGTCATCACCTTGGTGATG-CT
circAB-a-JR1-amplicon.seq(1>499)      →    GGGCGGTGTTGTCATAGCGACA-GTGATCGTCATCACCTTGGTGATG-CT
R-AD3-R-58_1.seq(8>437)               ←    GGGCGGTGTTGTCATAGCGACA-GTGATCGTCATCACCTTGGTGATG
R-ND3-R-59_1.seq(17>460)              ←    GGGCGGTGTTGTCATAGCGACA-GTGATCGTCATCACCTTGGTGATG-CT
AD1-R-48_1.seq(31>437)                ←    GGGCGGTGTTGTCATAGCGACA
R-ND3-F-50_1.seq(9>455)               →    GGGCGGTGTTGTCATAGCGACA-GTGATCGTCATCACCTTGGTGATG-CT
R-AD3-F-49_1.seq(9>440)               →    GGGCGGTGTTGTCATAGCGACA-GTGATCGTCATCACCTTGGTGATG-CT
AD1-F-45_1.seq(29>436)                →    GGGCGGTGTTGTCATAGCGACA-GTGATCGTCATCACCTTGGTGATG-CT
```

```
                                                    460       470       480       490       500
                                           GAAGAAGAAAC-AGTACACATCCATTCATCATGGTGTGGTGGAGATGAGC
circAB-a-JR1-amplicon.seq(1>499)      →    GAAGAAGAAAC-AGTACACATCCATTCATCATGGTGTGGTGGAGATGAGC
R-ND3-R-59_1.seq(17>460)              ←    GAAGAAGAAAC
R-ND3-F-50_1.seq(9>455)               →    GAAGAAGAAAC-AGTACACATCCATTCATCATGGTGTGGTGGAGATG
R-AD3-F-49_1.seq(9>440)               →    GAAGAAGAAAC-AGTACACATCCATTCATCATGGTGTGGTGGAGATGA
AD1-F-45_1.seq(29>436)                →    GAAGAAGAAAC-AGTACACATCCATTCATCATGGTGTGGTGGAGAT
```

```
                                                    510
                                           TGCTTC
circAB-a-JR1-amplicon.seq(1>499)      →    TGCTTC
```
