# Supplementary material for: Circular RNA Encoded Amyloid Beta peptides—A Novel Putative Player in Alzheimer’s Disease
Source: Cells. 2020 Sep 29;9(10):2196. doi: 10.3390/cells9102196 (PMC7650678; doi:10.3390/cells9102196)
Supplement: Supplementary file 1 [file cells-09-02196-s001.zip › revised supplementary data/Supplementary data-6-final.docx]

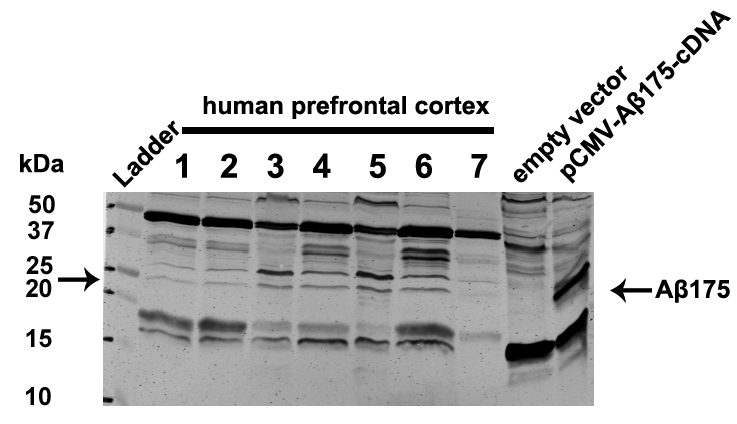


**Supplementary data-6. Western blot analysis of Aβ175 in human brain samples**

Ladder, protein size markers; empty vector, negative control vector transfection in HEK293 cells; pCMV-Aβ175-cDNA, HEK293 cells with pCMV-Aβ175-cDNA transfection; human prefrontal cortex 1-7, seven different human non-Alzheimer prefrontal cortex samples were used with an anti-Aβ175 antibody. As multiple bands have been observed in the blot, the specificity of this polyclonal antibody Anti-Aβ175 is low. The arrow shows a band in the expected size range of Aβ175 polypeptide, but the data are inconclusive.
