# Supplementary material for: Circular RNA Encoded Amyloid Beta peptides—A Novel Putative Player in Alzheimer’s Disease
Source: Cells. 2020 Sep 29;9(10):2196. doi: 10.3390/cells9102196 (PMC7650678; doi:10.3390/cells9102196)
Supplement: Supplementary file 1 [file cells-09-02196-s001.zip › revised supplementary data/Supplementary data-3-final.docx]

**Supplementary data-3**

**circAβ isoforms expression from the APP gene in human brain**

In order to experimentally analyze the generation of circRNAs from the Aβ region of APP gene, we resorted to RT-PCR amplification with a pair of specific divergent oligonucleotide (Aβ-VF2, Aβ-VR2 in Supplementary Fig. 1B); total RNA of human frontal lobe and hippocampus were used as templates. This design ensured that the amplified templates represented circular RNAs derived from the Aβ region of APP gene locus. Resulting PCR products were resolved by native 5 % polyacrylamide gel electrophoresis. Our analysis revealed that various different circRNAs are being generated from the Aβ region (Supplementary Fig. 1A). In order to investigate this pool of amplified circRNAs in more detail, we utilized deep sequencing of the corresponding PCR products and uncovered 16 distinct isoforms (Supplementary table-2). Amongst these circRNAs we recovered Hsa_circ_0007556[1], which is most the most abundant copy. For convenience, we refer it as circAβ-a (Fig. 1A, Supplementary table-2). Likewise, other additional circRNAs, were designated circAβ-b, -c, -d, …-q, respectively (Supplementary Fig. 1B, Supplementary table-2).

**
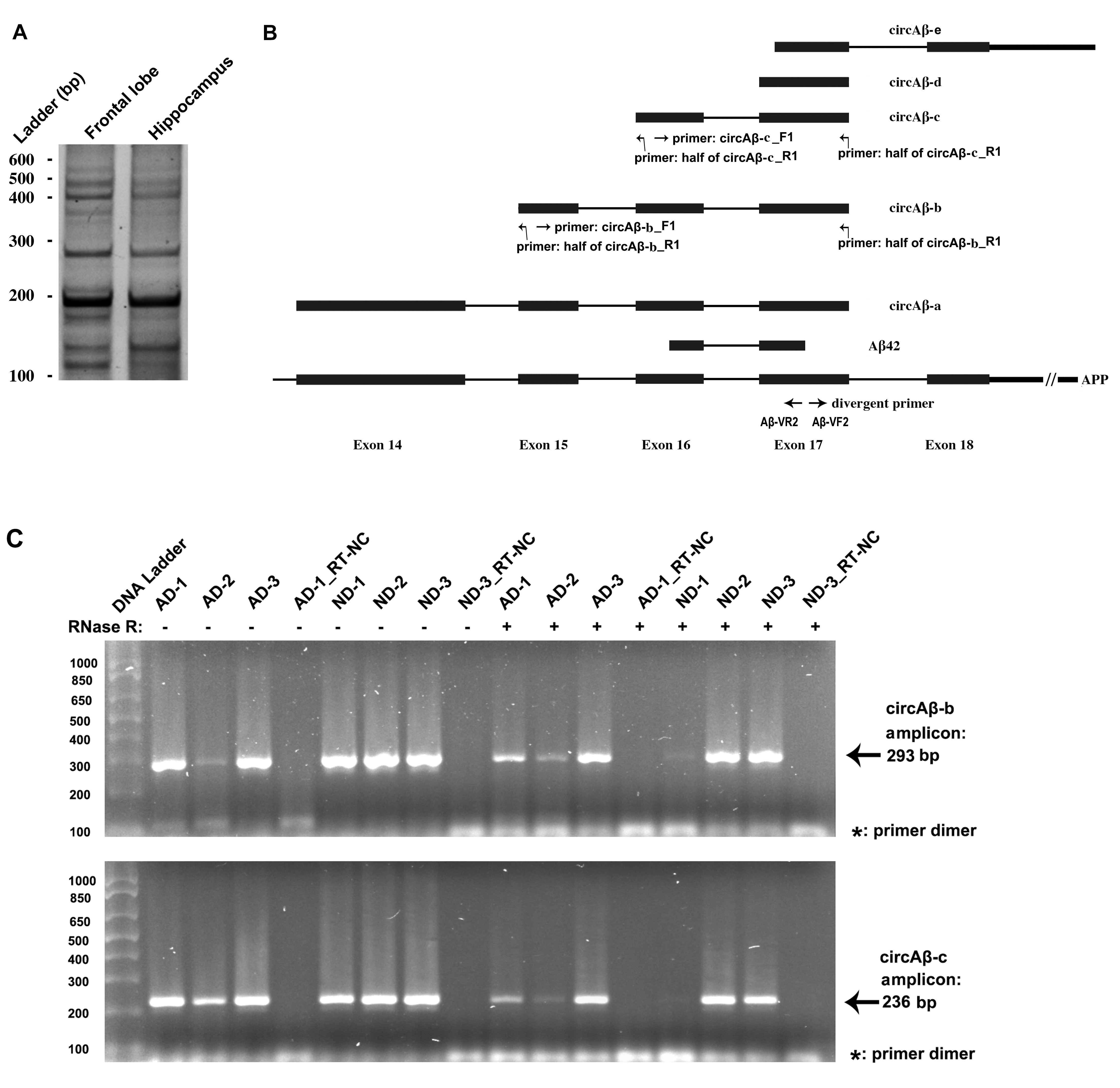
**

**Supplementary Fig. 1. The identification of circAβs in human brain in HEK293 cells**

**A. 5% native polyacrylamide gel electrophoresis of circAβ RT-PCR products** with divergent primer (Aβ-VF2, Aβ-VR2) located in the exon 17 of human APP gene in human brain samples; Two human brain RNA samples were used. **B. the localizations of circAβ-a, b, c, d, e in APP gene;** Aβ42 sequence was used as location reference. The amyloid-β (Aβ) sequence is located in exons 16 and 17; **C. RT-PCR verification of circAβ-a expression in the AD and nondementia (ND) control of human prefrontal cortex samples;** for RNase R treatment, total RNA was digested with 10 unites of RNase R for 1 hour at 37 °C; -, no treatment; +, with treatment.

**Confirmation of circAβ by RNA deep sequencing with total RNA enriched for circular RNA**

For independent verification of our sequencing analysis we repeated the circRNA identification with same total RNA that were RNase R pretreated (RNase R would digest linear RNA and leave circRNA unaffected). In summary, 15 out of 16 circAβs were resistant to RNase R treatment, indicating that they indeed represent circular RNAs. This second round of sequencing analysis uncovered one additional circAβ (Supplementary table-2). Importantly, circAβ-a was identified to be the most abundant copy (Supplementary table-2); this circRNA was 4.8/3.1-fold enriched within RNase R treated frontal lobe and hippocampus RNA samples (Supplementary table-2). We, therefore, selected circAβ-a as a model for our analysis of potential functions associated with circAβs.

| circRNA number | circRNA name | circAβ genomic  positions | Junction  reads of  sample 1 | Junction  reads of sample 2 | Junction  reads of sample 3 | Junction  reads of sample 4 | Size of spliced exons |
| --- | --- | --- | --- | --- | --- | --- | --- |
| **1** | **circAβ-e** | **chr21:25881397**  **-25891834** | **498** | **445** | **218** | **84** | **488 nt** |
| 2 | circAβ-f | chr21:25881459  –25891868 | 0 | 3 | 25 | 11 | 460 nt |
| 3 | circAβ-g | chr21:25881489  –25897673 | 21 | 3 | 37 | 1 | 531 nt |
| 4 | circAβ-h | chr21:25881523  –25897626 | 69 | 108 | 39 | 25 | 450 nt |
| 5 | circAβ-i | chr21:25881523  –25911815 | 12 | 13 | 0 | 0 | 626 nt |
| 6 | circAβ-j | chr21:25881537  –25891834 | 0 | 6 | 10 | 4 | 348 nt |
| 7 | circAβ-k | chr21:25881611  –25891834 | 17 | 20 | 27 | 19 | 274 nt |
| 8 | circAβ-l | chr21:25881611  –25891868 | 59 | 37 | 53 | 15 | 308 nt |
| 9 | circAβ-m | chr21:25881695  –25891855 | 0 | 0 | 20 | 8 | 211 nt |
| 10 | circAβ-n | chr21:25881695  –25897673 | 35 | 22 | 13 | 17 | 325 nt |
| **11** | **circAβ-d** | **chr21:25891722**  **–25891868** | **1519** | **643** | **755** | **571** | **147 nt** |
| **12** | **circAβ-c** | **chr21:25891722**  **–25897673** | **114** | **100** | **339** | **159** | **248 nt** |
| **13** | **circAβ-b** | **chr21:25891722**  **–25905077** | **104** | **88** | **218** | **139** | **302 nt** |
| **14** | **circAβ-a** | **chr21:25891722**  **–25911962** | **1464** | **1113** | **6965** | **3503** | **524 nt** |
| 15 | circAβ-o | chr21:25891733  –25897650 | 0 | 11 | 0 | 25 | 214 nt |
| 16 | circAβ-p | chr21:25891753  –25891834 | 0 | 51 | 0 | 23 | 82 nt |
| 17 | circAβ-q | chr21:25891753  –25897626 | 347 | 33 | 73 | 4 | 170 nt |

**Supplementary table-2. circAβ isoforms from the APP gene in human brain**.

circAβs genomic position and reads number from deep sequencing of RT-PCR products. The most enriched circRNA 14, 13, 12, 11, 1, are named as circAβ-a, b, c, d, e respectively (bold). Junction reads of sample 1: human frontal lobe total RNA without RNase R treatment. Junction reads of sample 2: human hippocampus total RNA without RNase R treatment. Junction reads of sample 3: human frontal lobe total RNA with RNase R treatment. Junction reads of sample 4: human hippocampus total RNA with RNase R treatment.

**Confirmation of circAβ-a, b, c isoforms expression within the AD and nondementia control brain samples by individual RT-PCR**

Finally, to confirm the individual expression of each circAβ-a, b, c isoforms within the AD and nondementia control of human prefrontal cortex samples, we performed RT-PCR analysis with human frontal lobe total RNA samples with oligonucleotide primers that designed to specifically detect circAβ-a, b, c (for details, Fig. 1A and Supplementary Fig. 1B, C). Indeed, circAβ-a, b, c were expressed both in within the AD and nondementia control of human prefrontal cortex samples (Fig. 1B, Supplementary Fig. 1C).

**Methods**

**CircAβ identification via RT-PCR**

Aβ containing circRNAs, which were derived from the APP (amyloid beta precursor protein) gene were amplified *via* RT-PCR with specific ‘divergent’, i.e., head-to-head oriented primers targeting protein coding exon 17 of the APP gene:

Aβ-VF2: 5’GTGATCGTCATCACCTTGGTGATGC3’

Aβ-VR2: 5’CACCATGAGTCCAATGATTGCACC3’

Two total RNA of human adult normal frontal lobe and hippocampus (R1234051-50-BC, R1234052-10-BC, BioCat GmbH) was used as template. cDNA synthesis was performed with SuperScript™ III First-Strand Synthesis SuperMix (18080400, Invitrogen) with random hexamers, according the manufactures’ recommendations. PCR was performed with PrimeSTAR® GXL DNA Polymerase (R050A, TaKaRa) with extension at 68 °C for 40 cycles with protocol provided by manufacture.

For enrichment of circular RNAs, 15 µg of total RNA of human frontal lobe and hippocampus were treated with 10 units of RNase R (RNR07250, Epicentre) for 1 hour at 37 °C and purified by phenol-chloroform extraction. 500 ng of the resulting RNA sample was utilized for subsequent cDNA synthesis and PCR amplification. PCR products were purified with QIAquick PCR Purification Kit according the manufactures’ recommendations.

**Deep sequencing and circRNA computational analysis**

RT-PCR products of CircAβs were used to prepare DNA sequencing libraries with the TruSeq DNA Nano Kit (FC-121-4003, Illumina, Inc) according the manufactures’ recommendations. All libraries were sequenced with the HiSeq4000 (Illumina, Inc) system at the Cologne Center for Genomics, University of Cologne. About one million reads were obtained for each sample and they were mapped with the STAR aligner (ultrafast universal RNA-seq aligner), then DCC (circRNA computational detection and quantification tool) for circRNA detection at the bioinformatics core facility of Max Planck Institute for Biology of Ageing [2,3].

**References**

1. Glazar, P.; Papavasileiou, P.; Rajewsky, N. circBase: a database for circular RNAs. *RNA* **2014**, *20*, 1666-1670, doi:10.1261/rna.043687.113.

2. Dobin, A.; Davis, C.A.; Schlesinger, F.; Drenkow, J.; Zaleski, C.; Jha, S.; Batut, P.; Chaisson, M.; Gingeras, T.R. STAR: ultrafast universal RNA-seq aligner. *Bioinformatics (Oxford, England)* **2013**, *29*, 15-21.

3. Cheng, J.; Metge, F.; Dieterich, C. Specific identification and quantification of circular RNAs from sequencing data. *Bioinformatics (Oxford, England)* **2016**, *32*, 1094-1096.
