# Supplementary material for: Circular RNA Encoded Amyloid Beta peptides—A Novel Putative Player in Alzheimer’s Disease
Source: Cells. 2020 Sep 29;9(10):2196. doi: 10.3390/cells9102196 (PMC7650678; doi:10.3390/cells9102196)
Supplement: Supplementary file 1 [file cells-09-02196-s001.zip › revised supplementary data/Supplementary data-4-final.docx]

**
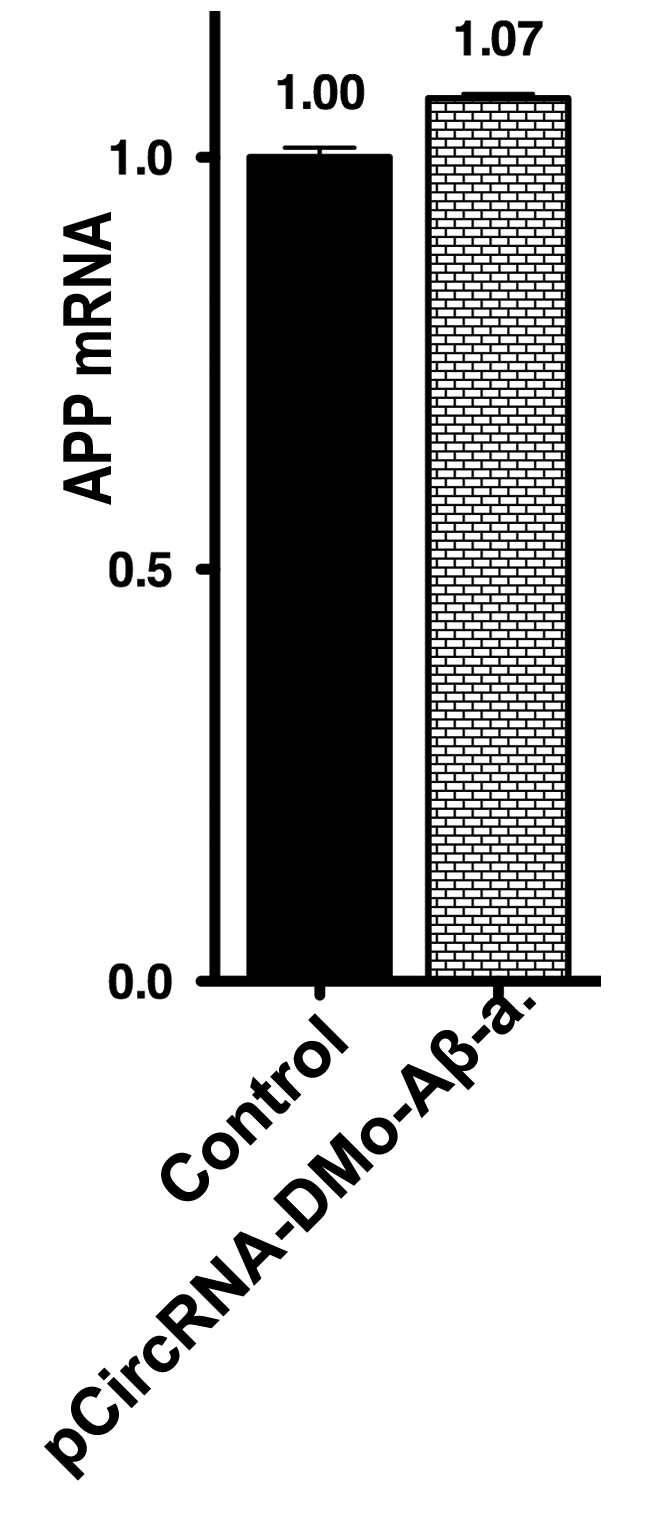
**

**Supplementary data-4. Endogenous APP mRNA expression in HEK293 cells overexpressing circAβ-a.**

qRT-PCR analysis of endogenous APP mRNA expression in circAβ-a overexpressing HEK293 cells. Primers targeted exons 3 and 4, which are not contained in circAβ-a: Control, pCircRNA-DMo empty vector, pCircRNA-DMo-Aβ-a, the circAβ-a expression vector. No significant differences between different groups were observed (n = 4).
